# Atomistic simulations reveal impacts of missense mutations on the structure and function of SynGAP1

**DOI:** 10.1093/bib/bbae458

**Published:** 2024-09-23

**Authors:** Aliaa E Ali, Li-Li Li, Michael J Courtney, Olli T Pentikäinen, Pekka A Postila

**Affiliations:** MedChem.fi, Institute of Biomedicine, Integrative Physiology and Pharmacology, University of Turku, FI-20014 Turku, Finland; InFLAMES Research Flagship, University of Turku, 20014 Turku, Finland; Neuronal Signalling Laboratory and Turku Screening Unit, Turku Bioscience Centre, University of Turku and Åbo Akademi University, Turku, Finland; Neuronal Signalling Laboratory and Turku Screening Unit, Turku Bioscience Centre, University of Turku and Åbo Akademi University, Turku, Finland; MedChem.fi, Institute of Biomedicine, Integrative Physiology and Pharmacology, University of Turku, FI-20014 Turku, Finland; InFLAMES Research Flagship, University of Turku, 20014 Turku, Finland; MedChem.fi, Institute of Biomedicine, Integrative Physiology and Pharmacology, University of Turku, FI-20014 Turku, Finland; InFLAMES Research Flagship, University of Turku, 20014 Turku, Finland

**Keywords:** SynGAP1, intellectual disability, structural bioinformatics, molecular dynamics (MD) simulation, in silico mutagenesis, missense mutation

## Abstract

De novo mutations in the synaptic GTPase activating protein (SynGAP) are associated with neurological disorders like intellectual disability, epilepsy, and autism. SynGAP is also implicated in Alzheimer’s disease and cancer. Although pathogenic variants are highly penetrant in neurodevelopmental conditions, a substantial number of them are caused by missense mutations that are difficult to diagnose. Hence, in silico mutagenesis was performed for probing the missense effects within the N-terminal region of SynGAP structure. Through extensive molecular dynamics simulations, encompassing three 150-ns replicates for 211 variants, the impact of missense mutations on the protein fold was assessed. The effect of the mutations on the folding stability was also quantitatively assessed using free energy calculations. The mutations were categorized as potentially pathogenic or benign based on their structural impacts. Finally, the study introduces wild-type-SynGAP in complex with RasGTPase at the inner membrane, while considering the potential effects of mutations on these key interactions. This study provides structural perspective to the clinical assessment of SynGAP missense variants and lays the foundation for future structure-based drug discovery.

## Introduction

SynGAP is a synaptic Ras/RapGTPase activating protein (GAP; UniProt: Q96PV0) [1] that is expressed abundantly at the postsynaptic density (PSD) of excitatory synapses in the forebrain neurons [2]. SynGAP negatively regulates the GTPase activity of small G proteins by accelerating their hydrolysis of guanosine triphosphate (GTP) into guanosine diphosphate (GDP). During long-term potentiation, SynGAP regulates the trafficking of α-amino-3-hydroxy-5-methyl-4-isoxazolepropionic acid receptors (AMPAR) to the PSD possibly involving the Ras signalling [1, 3, 4] and, afterwards, SynGAP is removed from the PSD through phosphorylation by calmodulin-dependent protein kinase II (CaMKII) in a N-methyl-D-aspartate receptor (NMDAR)-dependent manner [1, 5, 6]. SynGAP has been hypothesized to regulate how many PDZ-domain binding ‘slots’ are available at the PSD, which is needed for limiting the occupancy of the sites by the AMPAR-TARP (Transmembrane AMPAR Regulatory Proteins) complex, thereby modulating synaptic strength [5, 7–10]. Amyloid β-oligomers have been reported to prevent the accumulation of SynGAP at the PSD, where its loss is one of the most significant changes in an early stage tauopathy model [11]; accordingly, SynGAP may play a role in Alzheimer’s disease.

SynGAP-related non-syndromic intellectual disability (NSID) caused by de novo mutations of the *SYNGAP1* gene, leads to a variety of rare neurodevelopmental symptoms such as intellectual disability, autism spectrum disorder, and epilepsy [12–15]. These pathogenic variants of SynGAP account for 2%–8% of sporadic intellectual disability cases with >85% of patients suffering from epilepsy and ~ 50% are autistic [12, 14–18]. The numerous symptoms with varying severity become apparent in early childhood. The broad phenotypic spectrum of disorders presents a diagnostic challenge for clinicians due to the lack of specific clinical features [12, 19].

To date, only >1300 cases of SynGAP-related NSID have been diagnosed worldwide despite the higher expected incidence rate of 6 in 100 000 [20, 21]. Although most diagnosed SynGAP cases are gene truncations such as frameshift or nonsense mutations, several missense mutations, which simply replace an existing residue for another, are also known. The number of missense mutations submitted by clinicians into the ClinVar archive (N = 644, 22 April 2024) has been steadily increasing and so far, ~ 65% of them have been annotated as variants of uncertain significance (VUS; Fig. S1) [4, 19]. The status of any SynGAP missense variant is subject to change as conflicting patient data is constantly emerging. On a genome-wide scale, even as high as one-third of all missense mutations for proteins are estimated to be potentially pathogenic [22, 23]. For SynGAP, only half of the expected entries are present in the GnomAD (V4.0.0) database [24], which points to an intolerance to missense variation. The unclear pathogenic status of most missense mutations is keeping the number of diagnosed cases down, which risks lowering the interest and investment in drug development for SynGAP.

**Figure 1:**
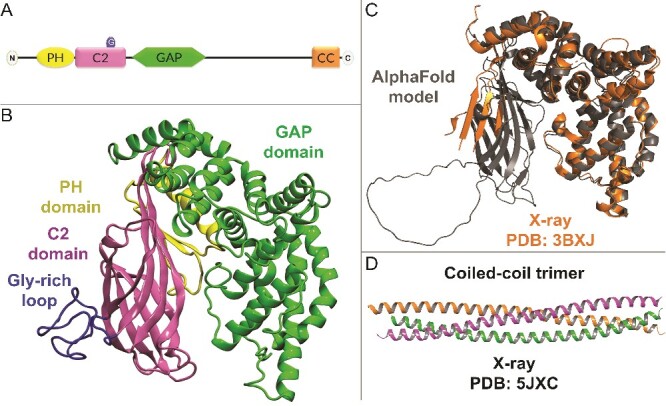
SynGAP domains and structure. (A) SynGAP is composed of PH (Pleckstrin homology), C2 (the second conserved PKC domain), Gly-rich Ω-loop motif (G), and GAP (GTPase activating protein) domains at the N-terminal region and the CC (coiled-coil) domain at the C-terminus. (B) Based on the pLDDT (predicted local distance difference test), the ≥50 reliable parts of the N-terminal region and less reliable Ω-loop from the human AlphaFold2 model are shown in 3D (cartoon model). (C) The X-ray crystal structure of rat C2-GAP fragment (orange cartoon; PDB: 3BXJ [26]) aligns well against the model (grey cartoon). (D) the X-ray crystal structure of the mouse CC trimer (PDB: 5JXC [27]) was not used in the modelling.

The N-terminal half of SynGAP consists of PH, C2, and GAP domains (Fig. 1A and B) that could be associated with the intracellular post-synaptic membrane. The C-terminal half consists of a 10 His repeat region, Ser/Tyr phosphorylation sites, a coiled-coil domain, and a T/SXV PDZ domain-binding motif that facilitates the postsynaptic scaffold protein interaction [5, 25]. The pathogenic missense mutations are distributed more on the known structural parts than the ‘disordered’ regions but, due to the prevalence of VUS, no other patterns can easily be recognized (Fig. S1). Only the C2-GAP fragment (Fig. 1C; PDB: 3BXJ [26]) and the coiled-coil trimer (Fig. 1D; PDB: 5JXC [27]) have been solved in 3D using X-ray crystallography. Hence, the lack of a proper 3D template has hindered the assessment of the SynGAP missense mutations at the structural level.

A workaround to the problem has been provided by AlphaFold2 – a revolutionary AI system from Google’s DeepMind [28]. It provides a workable 3D model for the N-terminal part of SynGAP, including GAP and C2 domains and most of the PH domain (Fig. 1B). Here, a comprehensive look is taken into the structural effects of the missense mutations using in silico mutagenesis, molecular dynamics (MD) simulations and folding free energy stability calculations (Table S1). The study also provides, to our knowledge, the first look into WT-SynGAP in complex with the intracellular post-synaptic membrane and its key interaction partner RasGTPase. Finally, the modelled variants are divided into potentially pathogenic (55%) or potentially benign (25%) categories based on the structural effects induced by the mutations. The remaining variants are given uncertain status (20%) despite their projected effects on complex formation with the membrane and RasGTPase or due to their proximity to the model termini which lack reliable coordinates.

Although drug repurposing screens have found preliminary hits (e.g. anti-inflammatory drugs [29]), there is no effective treatment for the SynGAP-related NSID. By examining SynGAP with a structural focus, this study paves the way for structure-based drug discovery as well as improving the diagnosis of the missense variants.

## Materials and methods

### In silico mutagenesis and molecular modelling

**Figure 2:**
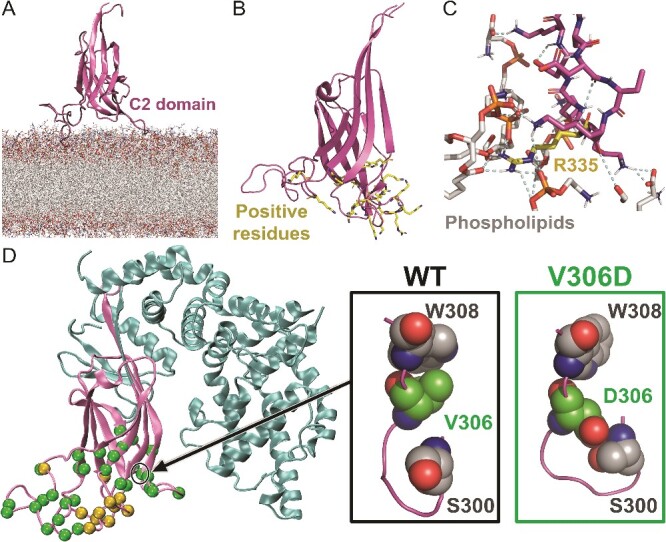
The C2 domain of SynGAP at the membrane surface. (A) At 300 ns, the C2 domain of WT-SynGAP (cartoon model) is shown stably standing via its ‘loop legs’ at the surface of the inner leaflet membrane model (stick representations). (B) The C2 domain of SynGAP is shown to have positive residues at the interface of the inner leaflet membrane. (C) A zoom-in highlights the hydrogen bond (or H-bonds) and salt bridges formed by Arg335 (orange stick model), explaining the pathogenic potential of the R335H variant when the positive charge is lost and/or bonding network is altered. (D) On the left, those missense mutation residue positions that are at the membrane interface and directly involved in H-bonding are highlighted with green- and orange-coloured spheres, respectively. On the right, a zoom-in shows Val306 packing against Trp308 (CPK models) for the WT system at 50 ns. The V306D mutation disrupts this interaction to allow H-bonding with Ser300 at the membrane-facing loop.

**Figure 3:**
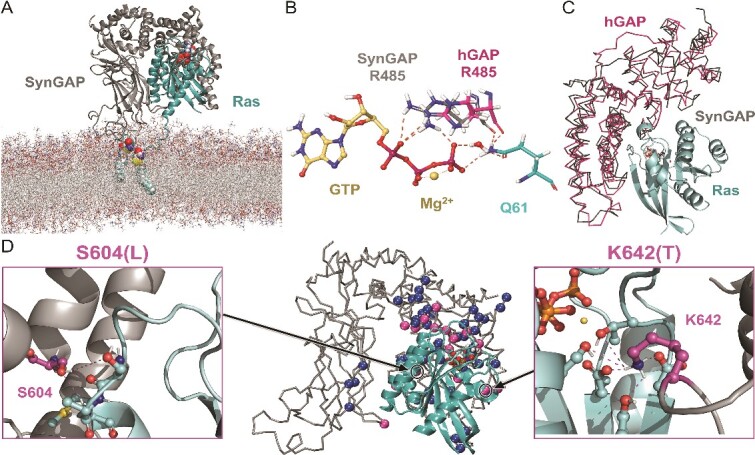
The SynGAP-RasGTPase complex at the inner postsynaptic membrane surface. (A) At 130 ns, the WT-SynGAP-Ras complex (cartoon model) is shown at the inner leaflet membrane model (stick representations) with the bound GTP molecule (CPK model). Covalently linked Cys-palmitoylated lipids 181 and 184 anchor Ras to the membrane (CPK). (B) A zoom-in shows the Ras-bound GTP-Mg^2+^ complex (ball-and-stick models), interacting with the catalytic arginine finger or Arg485 of SynGAP and Gln61 of Ras (stick models). (C) For reference, the human GAP-Ras complex (pink ribbon model; PDB: 1WQ1 [37]) is shown aligned with the SynGAP model (grey ribbon model). (D) The missense mutations directly at the GAP-Ras interface or close-by are shown as pink spheres (C^α^ atoms) and the ones H-bonding with Ras as blue spheres. The close-ups show SynGAP residues Ser604 and Lys642 H-bonding with Ras, accordingly, their mutagenesis to leucine or threonine, respectively, could weaken the GAP-Ras association.

The AlphaFold model for SynGAP α2 isoform-1 (UniProt: Q96PV0–01; AF-Q96PV0-F1-model_v3.pdb; https://alphafold.ebi.ac.uk/; 22 January 2022) [28]) was edited to contain reliable (pLDDT ≥50) N-terminal region and the Gly-rich loop (res. 198–730; Fig. 1B and C). Altogether 204 missense variants from ClinVar (https://www.ncbi.nlm.nih.gov/clinvar/; 21 April 2023) [30] and seven from other sources (e.g. MASTERMIND; https://mastermind.genomenon.com/; 21 April 2023) [31] were generated using MODELLER10.3 [32] (Table S1). SynGAP-membrane positioning (Fig. 2A and Fig. S2A) was acquired by aligning the Type II C2 domain against a Protein Data Bank-entry (PDB; https://www.rcsb.org/) [33] with settled C2 domain-membrane orientation at the Orientations of Proteins in Membranes (OPM; https://opm.phar.umich.edu/; 11/10/2022; PDB: 4RJ9) [34, 35] using VERTAA in BODIL [36] (Fig. S2B–C available online at http://bib.oxfordjournals.org/). SynGAP-RasGTPase complex was modelled by aligning the GAP domain with a human GAP-RasGTPase structure (PDB: 1WQ1; Fig. 3A and B) [37], and, at the active site, the GDP-AlF_3_ was converted to GTP in MAESTRO (Schrödinger Release 2022–3: Maestro, Schrödinger, LLC, New York, NY, 2022). A more detailed description is given in the Supplementary Information (SI).

### Molecular dynamics simulations and protein stability calculations

SynGAP-solvent systems were prepared for the WT protein (Fig. 1B) and missense variants with TLEAP in AMBERTOOLS22 [38] and simulated using AMBER22 [39], each running for 3 x 150 ns. The AMBERff19SB and OPC force fields were used for the protein and solvent, respectively [40, 41]. The SynGAP-membrane (Fig. S2A), SynGAP-RasGTPase-membrane complex (Fig. 3A) and intracellular membrane only [42, 43] systems were modelled using CHARMM-GUI and MD simulated 130, 300, and 150 ns, respectively, using GROMACS2021 [44] and CHARMM36ff [45]. Ras was Cys-palmitoylated at residues 181 and 184 (Figs. 3A and Fig. S3). Protein folding stability calculations for the variants were performed using FoldX5.0 [46] based on frames from the 150-ns WT-SynGAP-solvent simulations [47] and, likewise, using Rosetta Cartesian ΔΔG protocol [48–54]. Protocols (Fig. S4) and input files (supplementary_files.zip) are given in the SI.

### Analysis and figure preparation

The MD trajectories were examined, and figures were generated using MOL^*^4.0.1 [55], VMD1.9.4a12 [56], PYMOL2.3 [57], and MAESTRO. The trajectory analysis, including Root-Mean-Square-Deviation (RMSD; Figs. S5–S6) and atomic fluctuation (Fig. S7) calculations [58], was performed using CPPTRAJ in AMBERTOOLS22 [38]. With SynGAP-membrane simulations, the lipid bilayer metrics were analysed using GROMACS2021 [44] tools (Fig. S8).

## Results

### Resilience of the SynGAP model in simulations

The SynGAP-solvent MD simulations, probing the structural effects of the missense mutations (N = 211; Table S1), indicate that the AlphaFold2-derived model (Fig. 1C) is resilient to large-scale changes due to mutagenesis. This stability is apparent when comparing the Ramachandran plots for the WT and variant simulations (Fig. S9A-F) [59]. The normalized B-factor values [58] indicate that most of the movement happen in the unstructured loops regardless of the system (Figs. S7 and S10). A re-adjustment can happen to accommodate the mutation; however, typically the RMSD curves level up at the end (Fig. S5).

**Figure 4:**
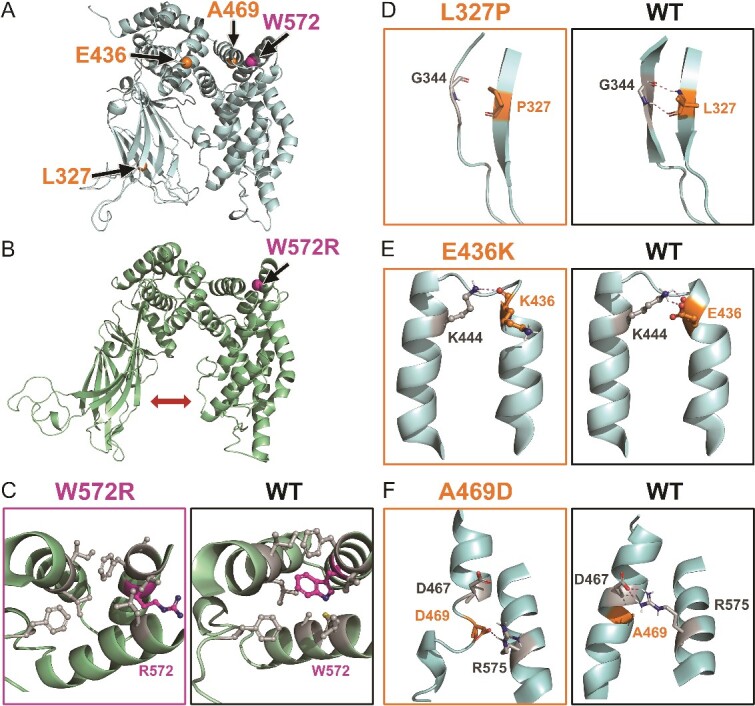
SynGAP missense mutations disrupting secondary structure, tertiary structure bonding and causing inside-out disruptions. (A) The positions of potentially pathogenic mutations L327P on the β hairpin (res. 322–349), E436K on the α helix (res. 414–436), A469D on the α helix (res. 461–476), and W572R on the α helix (res. 563–578) are shown as spheres on the SynGAP model structure (cartoon model). (B) The W572R variant at 150 ns of the second replicate simulation shows the C2 and GAP domains moving away from each other (arrow). (C) A close up of the W572R variant shows how the positively charged arginine is unable to remain in the hydrophobic inter-helix space as was the case with the original tryptophan. (D) In the L327P variant, the introduced proline cannot maintain the same hairpin backbone H-bond with Gly344 as the leucine does in the WT protein. (E) In the E436K variant, when the negatively charged glutamate is substituted with the positively charged lysine, the salt bridge with Lys444 is exchanged for a charge repulsion that breaks the parent α helix (res. 440–458). (F) the Ala469 resides in a hydrophobic inter-helix space in the WT protein, but, in the A469D variant, the negatively charged aspartate escapes to the outer surface of the α-helix and forms a salt bridge with Arg575. The zoom-in views (D, E, F) were generated using snapshots captured at the 50 ns timepoints in the MD simulations. The mutated residues are shown as ball-and-stick models.

A notable exception to this stability was the W572R variant, which underwent a major rearrangement in one of its replica simulations (Fig. 4A-C). Likewise, large-scale instability was induced with the WT system, when it was simulated at an elevated temperature (310 K → 400 K; Fig. S11). The truncated PH domain began to break its intra-protein interactions and rotate after ~20 ns in all three 400 K replica simulations. Because this ‘unfolding’ event could be repeated and it happened at the truncated domain, lacking the structural integrity to withstand the temperature boost, this was not studied further. Likewise, the simulation outcomes should not be considered reliable for the residues close to the model termini (‘Uncertain’; Table S1).

Although the missense mutations did not cause large-scale unfolding, they did cause notable conformational changes near the mutated residues (Figs. S5, S7, S9, S10). The residue swaps could affect the hydrophobic packing, disturb the intra-protein H-bonding network as well as weaken the secondary or tertiary structure. The variants were grouped into potentially benign (N = 52), potentially pathogenic (N = 117), and uncertain (N = 42) categories (Table S1). If the mutation was introduced near one of the truncated protein ends, the variant was given an uncertain status. No variant was assigned pathogenic based on its modelled location at the GAP-Ras or C2-membrane interfaces, unless the mutation had an adverse effect otherwise. A warning was given when there was a clear disparity between the expected impact and the simulation outcome (‘MD alert’; Table S1).

### Mutations disrupting the secondary structure

Several missense mutations disrupt secondary structure elements in the SynGAP model based on the MD simulations (‘Secondary’; Table S1). Although these effects were often minor on the overall fold, due to the integral role of α helices and β sheets for the protein’s structural unity or the underlying folding process, the actual mutagenesis effects are potentially pathogenic [23, 60–64]. It is noteworthy that a secondary structure element can tolerate up to 20% of the ‘breaker’ residues (Gly, Pro, Ser, Asn, and Asp), and thus, a cluster of breakers can be needed to convert elements to loops or turns [65].

The negative effects on the secondary structure are straightforward with proline substitutions (Fig. 4D and Fig. S12A-B). Proline lacks the backbone amide needed for the H-bonding between the peptide bonds in the secondary structure elements [66–68]. In total, 14 proline variants, which clearly affect the integrity of secondary elements (e.g. R544P, L465P, and L431P), could be recognized. For example, in the L327P variant, the proline swap removes the backbone H-bond with the carbonyl of Gly344 at the middle of an anti-parallel β sheet in the C2 domain causing it to unfold (Fig. 4D). Likewise, with the L431P variant, the proline is introduced in the middle of an α helix, which removes the backbone H-bond with the carbonyl of His427 and leads to the helix breakage in the GAP domain (Fig. S12B).

Conversely, the rigid cyclic structure of proline facilitates tight peptide chain turns and limits the continuity of α helices and β sheets [69]. Although the proline replacements can affect the folding profoundly (e.g. P349S, P562L, P605R, and P605S), they did not generate drastic unfolding effects in the simulations. Similarly, glycine, second only to proline in low helix propensity [70], can transform an α helix into a loop due to its flexibility [71]; however, no such disruptions were visible (e.g. E419G, A541G, E496G, W572G, A577G, S590G, and E666G). Additionally, the serine swaps are known to generate bends or destabilize α helices, due to its sidechain hydroxyl H-bonding with the backbone atoms of the neighbouring residues [65, 66, 72]. Nevertheless, these ‘breakers’ did not affect the helix continuity (e.g. F420S, I510S, W572S, and L664S).

The β-hairpins, which consist of two antiparallel strands coupled by a turn and technically belong to super secondary structure motifs, have been proposed to act as nucleation sites for protein folding [73–78]. During annotation, missense mutations disrupting the β-hairpins were considered potentially pathogenic (e.g. L274Q, D287H, R299C, and L327P). The β-hairpins are abundant in the C2 domain that is composed of a β-sandwich (Fig. 1B). While in a few cases, the negative effect of the mutation on the hairpin was apparent (e.g. L327P; Fig. 4D), typically, it was difficult to ascertain their importance relying solely on the simulation outcomes.

### Mutations affecting tertiary structure bonding

The SynGAP missense mutations that affect weak bonding interactions between domains or secondary structure elements, including flexible loops and turns, were considered as tertiary bonding effects (‘Tertiary bonds’; Table S1). The focus was given on gaining or losing H-bonds and salt bridges, because they can act as indicators of larger tertiary structure changes. Salt bridges and long-range electrostatic interactions are important for folding, facilitating tertiary assembly, and for improving the thermal stability [79–82]. The tertiary structure formation represents the final stages of the folding process [83], and, therefore, the variant simulations provide a viewpoint to the tertiary effects.

Indeed, several of the mutations were determined to affect the tertiary bonding or assembly based on the simulations (e.g. R342W, R405C, R429W, E436K, R573L, R596H, E666G, and R259W; Fig. S12C). For example, in the WT protein, Glu436 forms a salt bridge with Lys444 – a tertiary arrangement keeping two or even three α helices in contact. In the E436K variant, this inter-helix interaction is reversed by substituting the negative glutamate with a positive lysine that is repelled by the equally positive Lys444. This results in the shortening of the Lys444-containing helix and a compromised tertiary assembly (Fig. 4E). As a rule of thumb, when a charged group becomes either unpaired or wrongly paired via mutagenesis, as is the case with the E436K variant, misfolding can follow [65].

### Inside-out mutations: Solvent exposure of buried residues

A missense mutation can render a buried residue unfit for its native intra-protein environment Table S1), which then leads to inside-out effects expulsing and/or misplacing sidechains. Because the hydrophobic effect is one of the major driving forces behind the protein folding [84], these inside-out effects can be detrimental to establishing a functioning protein. For example, with the C2 domain the inside-out mutations have a clear negative effect on the entire β-sandwich structure, although no specific bonds are necessarily altered near the mutation site (e.g. L264Q).

Mutations altering hydropathy, charge, and/or size, may expel the mutated sidechain (e.g. L323R, G390E, L402R, and A469D; Fig. 4F) or nearby residues from the protein interior (e.g. I494R, C547R; Fig. S12D-E). For example, the A496D variant introduces a negatively charged sidechain into the hydrophobic inter-helix space. The carboxylate of aspartate moves to the out-facing side of the α helix and forms a salt bridge with the guanidium of Arg575 to escape the electrostatically unfavourable environment, which, in turn, breaks the parent helix. Likewise, the I494R variant introduces a positive sidechain into a hydrophobic inter-helix space, where there is not enough room nor accommodating charge environment, which results in the loss of integrity of the α helix facing the introduced arginine.

**Figure 5:**
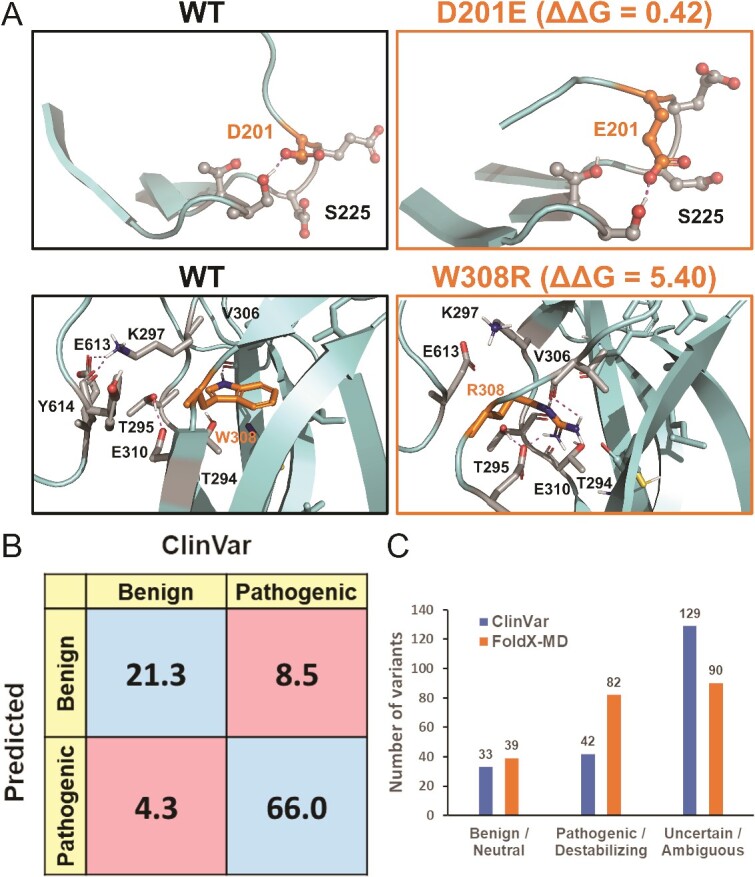
Protein folding stability calculations. (A) At 150 ns, Asp201 and Glu201 (stick models) at the antiparallel β sheet strand (res. 205–208) in both the WT and benign D201E variant simulations, respectively, H-bond with Ser225. The lack of adverse simulation effects is matched by the neutral ΔΔG value (0.42 kcal/Mol) in the FoldX-MD calculations. Conversely, the W308R variant introduces a positive arginine into an anti-parallel β sheet strand (res. 305–315) and within the C2 domain’s hydrophobic core. Although impossible from the folding point of view, the mutation causes only structural distortion without large-scale unfolding in the MD simulations. In contrast, FoldX-MD calculations give the W308R variant a strongly destabilizing ΔΔG value (5.40 kcal/Mol), highlighting its pathogenic status. (B) The confusion matrix compares the neutral and destabilizing predictions of the FoldX-MD approach against the benign (or likely benign) and pathogenic (or likely pathogenic) variants of ClinVar. The percentage of the true positives and negatives are in blue, while the percentages of the false positives and negatives are in red. (C) The number of neutral predictions is only slightly higher than the number of known benign variants, nonetheless, more variants are deemed as destabilizing than have been assigned as pathogenic. The conflicting variants E310K, P562L, and R573W were included as likely pathogenic and R575C as likely benign based on their most prevalent submitted status.

Although the inside-out mutations are likely deleterious, typically no large-scale unfolding was seen in the simulations (e.g. W308R, C282R, and A271D; Fig. 5A and Fig. S12F, S12G). An exception to this was the W572R variant, where the expulsion of positive arginine from the inter-helix space caused the GAP and C2 domains to move apart (Fig. 4B and C); however, this unfolding event was not replicated.

### SynGAP-Ras complex formation at the inner leaflet of the synaptic membrane

The WT-SynGAP-RasGTPase complex was modelled at the inner post-synaptic membrane based on four criteria to scrutinize its molecular interactions:

1. The C2 domain of SynGAP was determined to have Type II β sheet arrangement based on structural comparison against known Type I and II examples (Fig. S2B). The Type II C2 domains typically interact with the membrane surfaces in an upright position rather than via the sides, front or back of the β-sandwich fold [25]. Hence, the PH domain is not suggested to interact directly with the membrane, which is supported by the fact that most PH domains are not capable of independent membrane targeting [85].
2. The upright positioning of the SynGAP-C2 domain at the membrane surface was acquired by aligning it on top of the C2 domain of another protein (Fig. S2C) with settled membrane positioning [34, 35]. Notably, during the 150-ns WT-SynGAP-membrane simulations, the C2 domain remains stably membrane-bound, upright, and standing on ‘loop legs’ at the membrane surface (Fig. 2A).
3. The selected upright C2 domain positioning allowed several positive residues to face the polar or charged phospholipid head groups of the lipid bilayer in the SynGAP-membrane simulations (Fig. 2B and C; Table S2). Electrostatic forces have been suggested to be the main driving force behind the binding of Ca^2+^-independent C2 domains into membranes [25]. This not only supports the validity of the selected SynGAP-membrane assembly, but it also provides support to the notion that the binding of SynGAP on the inner membrane would follow the Ca^2+^-independent mechanism.
4. Lastly, the upright SynGAP-C2 domain positioning at the membrane is ideal for the GAP domain to bind unobstructed with its key interaction partner RasGTPase (Fig. 3A). The catalytic arginine finger of GAP binds to the Ras-bound substrate GTP (Fig. 3B) in the modelled SynGAP-Ras complex similarly as the equivalent domain of human GAP-Ras complex (Fig. 3C) [37]. The covalently linked Cys-palmitoylated lipids of Ras can easily dip into the inner membrane leaflet in this spatial arrangement. Ras has been postulated to have several different membrane orientations [86–91], it could even be adjusted by the nucleotide binding [88, 90], however, during dimerization [86] or when Ras is complexing with Raf [90, 91], a similar orientation has been suggested. Importantly, the WT-SynGAP-Ras complex was stably formed and membrane-bound in the simulation.

### Mutations potentially altering the C2 domain-membrane association

From the perspective of the membrane-bound WT-SynGAP model (Fig. S2A), the C2 domain missense mutations – particularly those altering the membrane-facing loop dynamics (Table S1) – may compromise the stable SynGAP-membrane association. Here, the C2-membrane interaction was only probed with the WT protein; however, some interface mutations already displayed effects in the solvent simulations.

For example, with the A271D variant, the carboxylate of the aspartate is introduced into the C2 domain’s hydrophobic niche and, consequently, it forms H-bonds or salt bridges with the nearby loop residues (e.g. Lys394), which could decrease the flexible loop movement needed for the dynamic C2-membrane association (Fig. S12G). Likewise, with the V306D variant (Fig. 2D), the negative aspartate is repelled by the native hydrophobic partners of the WT residue valine (e.g. Trp308) and attracted by a new set of hydrophilic partners (e.g. Ser300). In general, the Ω-loops are known to have roles in protein functions mandating flexibility, and, thus, they rarely contain hydrophobic residues [92, 93]. Accordingly, the multiple valine substitutions into the Gly-rich Ω-loop motif of SynGAP (e.g. G373V, G381V, G387V, and G391V; Figs. 1B and 2D and Fig. S6) could reduce its flexibility, which, hypothetically, is needed for accomplishing the stable membrane association.

In theory, mutations that induce structural strain within the C2 domain (N = 85; Fig. S1) or disturb its direct interactions such as H-bonding with the membrane (Table S2; Fig. 2D), could compromise membrane binding and prove to be pathogenic. Nevertheless, in the absence of definitive evidence, these variants were only itemized, if the mutated residue was proximal to the membrane in the WT-SynGAP-membrane simulation.

### Mutations potentially affecting the SynGAP-Ras association

The binding of the GAP domain to the RasGTPase (or RapGTPase) is a key effector interaction of SynGAP (Fig. 3A). Notably, its arginine finger (Fig. 3B) promotes the hydrolysis of GTP to GDP at the active site of Ras [61]. Although only the WT-SynGAP-Ras complex was simulated, from this perspective, any mutations directly at the protein–protein interface (Fig. 3D), for example, participating in the SynGAP-Ras H-bonding (Table S2; Fig. 3D) or relaying an allosteric effect from further away could perturb the control of GTPase activity.

There exists at least one missense mutation categorized as pathogenic (W362R; Table S1) and several VUS at the GAP-Ras interface that could weaken or even disrupt the protein–protein association (e.g. S604L, K642T, and R596C; N = 24; Table S1 and Table S2). For example, the hydrophobic sidechain of leucine in the S604L variant cannot H-bond with either Ras residue Ser65 or Ala66 similarly as the hydroxyl-containing serine of the WT protein. Likewise, in the K642T variant, the salt bridge formation between the WT residue lysine and the carboxylate groups of Asp33 and Asp38 at the Ras side is prevented by the residue substitution (Fig. 3D).

Because the connection between the compromised Ras activity and the SynGAP-related NSID is uncertain [6], variants that could alter the protein–protein interactions were only itemized (‘GAP-Ras interface’; Table S1) and given uncertain status (Table S1).

### No structural effects: Potentially benign missense mutations

Plenty of SynGAP missense mutations have been categorized as harmless (N = 111; Table S1). While this benign or likely benign status can change due to emerging patient data, the MD simulations also suggest that many of the mutations are well-tolerated. Here, we considered a missense mutation potentially benign when it did not cause any apparent structural change, the effect was only minor, or its undesirable nature was unclear. For example, substituting valine with leucine (e.g. V400L; Fig. S12H), even when the sidechains are facing inward, the structural stability was not visibly affected during the simulations. A fraction of the missense mutations that were given the potentially benign status, are located at the protein surface, where they are unlikely to affect the fold or interact at least with the membrane or RasGTPase (e.g. A433V, V441A, L655Q, and W697R). Despite the uncertain status, several variants at the truncated model termini could be benign as well (e.g. D201E; Fig. 5A).

### Pathogenic mutations destabilizing the structure

To complement the MD-based annotation, a quantitative modelling method was applied to explore the missense effects on the protein folding (Fig. 5B and C; Table S1). The FoldX-MD protocol [47], in which the mutations are assessed in the context of multiple WT simulation frames, is widely used to identify how single-point mutations affect the folding stability [47, 94–98]. The calculations predicted that 82 mutations destabilize the protein (e.g. C282R, W308R), 31 have a neutral effect (e.g. T532P, S535T), 89 fall into the ambiguous category, while none were predicted as stabilizing (Fig. 5C). The predictions should be considered unreliable for the PH region lacking structural integrity (Fig. S11). Although the calculations showed an 85% agreement with the MD-based annotation, a few differences were observed (e.g. D287Y, S300F, R429W, L465V, and G580S).

The folding stability calculations were useful in pointing out potentially pathogenic effects for the following mutation types:

1. The introduction of charged and bulky residues into the hydrophobic intra-protein positions was predicted to be strongly destabilizing (e.g. W308R; Fig. 5A).
2. Mutations that introduced a small polar residue like serine into the hydrophobic protein regions resulted in destabilizing energies (e.g. F420S, I510S, W572S, and L664S). Similarly, mutations that replaced a large hydrophobic residue with a smaller polar one, such as cysteine, were found to be destabilizing, in part due to the sidechain size difference (e.g. Y497C, W308C).
3. Variants that introduced proline within secondary structure elements were clearly unfavourable based on both the MD simulations and stability calculations (e.g. R293P, L323P, L431P, and L327P; Fig. 4D). However, the removal of proline from the ends of secondary structure elements via residue substitutions was also considered firmly destabilizing by the FoldX-MD calculations (e.g. P349S, P562L, P605R, and P605S) [67–69].
4. Residue swaps that introduced valine into the flexible Ω-loops were flagged as highly destabilizing (e.g. G373V, G381V, G387V, and G391V). The potential pathogenic effects of these mutations are understandable, provided that the C2-loops are in dynamic interaction with the membrane (Fig. 2A-C).

The FoldX-MD results were compared against variants with currently agreed upon non-VUS or non-contradictory status in the ClinVar to find out if the stability predictions matched the clinical data (Fig. 5B). Overall, the ΔΔG values indicate that the pathogenic (N = 11) or likely pathogenic variants (N = 31) were more often predicted to be destabilizing (e.g. W308R) than their benign (N = 17) or likely benign (N = 15) counterparts (e.g. D201E; Fig. 5A). Although this data set is limited (N = 74) and, by no means, should the status of the variants be considered settled, the comparison indicates an accuracy of ~ 87% Table S3) for the FoldX-MD calculations.

Because FoldX tends to predict stabilizing mutations with low accuracy [94, 99], Rosetta was also used to calculate folding energy [50, 99, 100]; however, it did not find any stabilizing mutations either (Table S1 available online at http://bib.oxfordjournals.org/). Rosetta had prediction accuracy of ~ 80% (Table S3 and Fig. S13A). Furthermore, Rosetta was more prone to assign benign status, while FoldX-MD was more effective at recognizing pathogenic mutations (Fig. 5A and Fig. S13A and Table S3). The methods showed ~ 73% overlap (Fig. S13D).

## Discussion

The missense variants within the scope of the N-terminal SynGAP model (Fig. 1B; N = 211; Table S1) were in silico-generated and MD simulated. Although the residue substitutions did cause notable local changes, overall, the protein fold was resilient to change. However, because the simulations are not addressing the folding problem [101], they cannot account for the effects that the missense mutations exert already during translation and folding. The modelling approach, applied in multiple studies [64, 102–116], works in a backwards manner; i.e. the mutations are introduced to the already folded protein. For example, although the hydrophobic effect is a major driving force in folding [84], the introduction of charged residues into the hydrophobic intra-protein positions did not typically cause large-scale unfolding (e.g. W308R; Fig. 5A). An exception to this trend was the W572R variant, where the GAP and C2 domains moved apart due to the inside-out mutation in one of the replicate simulations (Fig. 4B and C).

The conformational resilience or stability makes sense from the MD perspective: the hydrophobic intra-protein regions are fitting for neither the solvent nor the charged residue, but large-scale unfolding would not be an energetically favorable option either. To provide a quantitative picture of the structural effects of the missense mutations, protein folding stability calculations were performed. FoldX is regarded as one of the most accurate single site mutation stability predictors [94, 98, 99] and the FoldX-MD workflow further improves its accuracy [47, 94, 95, 117]. This approach was effective in recognizing even those likely pathogenic variants that showed muted effects in the simulations (‘MD alert’; Table S1). Here, the Rosetta calculations yielded a similar overall picture (Table S1; Fig. 5A and Fig. S13A).

Nevertheless, experimental evidence on the SynGAP missense variants suggests that instability is not a major factor for the pathogenicity [4]. Only a few variants, including W362R, R573L, P562L, and T790A, have been experimentally linked to structural instability so far [4]. The robustness of the fold could be attributed to its size (140 kDa; 1343 aa), which provides a shielding factor to heating-induced instability and unfolding [4, 12, 102]. The size argument does not explain the resilience seen in the simulations, because just the monomeric N-terminal section was studied (60 kDa; 533 aa; Fig. 1B). The nuanced structural effects of the missense mutations as evidenced in the simulations could be enough to cause the disease phenotype similarly as the truncating mutations. Indeed, based on a limited medical data analysis, missense mutations cause similar disease phenotypes as truncating, intronic, and microdeletion mutations [118].

By exploring WT-SynGAP in complex with the membrane (Fig. 2), it becomes evident that most of the C2 domain missense mutations, especially in the membrane-facing loops, could be pathogenic (e.g. V306D; Fig. 2D; ‘At membrane’; Table S1). While the membrane proximity was not used as a criterion in assigning pathogenicity, the membrane-facing mutations could affect the strength, coordination, flexibility, and/or stability of the SynGAP-membrane association [23, 119, 120]. The C2 domain is not tethered to the intracellular membrane leaflet via covalently attached lipids as is the case for the RasGTPase (Fig. 3A). Hence, variants disrupting the C2 domain structure, such as L327P (Fig. 4D), could affect its ability to bind onto the synaptic or intracellular membranes. An indirect effect of the C2 domain misfolding, or its altered membrane adherence, on the SynGAP function could be its inability to regulate AMPAR membrane trafficking and/or its increased difficulty of interacting with the GTPases. Interestingly, the C2 domain greatly accelerates activity of both Rap- and RasGTPases in cell-free assay lacking the membrane component [26], which suggests that, regardless of the membrane, the catalytic activity depends on the presence of both C2 and GAP domains.

The modelling of the WT-SynGAP-RasGTPase-membrane complex shows that multiple missense mutations are located at the protein–protein interface, affecting for example inter-protein H-bonding (Fig. 3D; Table S2). Changes at the interface could prove to be disease-causing as the dysfunction of Ras has been linked to several diseases affecting the nervous system (e.g. Alzheimer’s disease), neuroinflammation and even brain tumors [121–126]. Notably, *SYNGAP1*, also known as *RASA5* (Ras GTPase-activating protein 5), functions as a tumour suppressor that downregulates the catalytic activity of Ras, which, in turn, inhibits the epithelial-mesenchymal transition and metastasis [127]. Regardless, a recent study suggests that the catalytic activity of Ras, broken by the R485L/F484A mutation would not result in the SynGAP-related NSID phenotype nor prenatal death with mice despite the significantly reduced Ras activity [6]. Indeed, the R485C variant has not been diagnosed as pathogenic (Table S1). Hence, the placement of the mutation at the GAP-Ras interface was not used as a criterion for pathogenicity.

Even if the regulation of catalytic activity of the Ras-family GTPases is not responsible for the SynGAP-related NSID phenotypes [6], there may still be a functional role for the SynGAP-Ras interaction. Sufficient association could be needed for the membrane binding or scaffolding. The Ras-family GTPases reside on the membrane on a more permanent basis than SynGAP due to the Cys-lipidation (Fig. 3A) [128, 129]. In theory, the lipid anchors of Ras (e.g. palmitoyl, farnesyl) could assist in the SynGAP localization to a certain lipid raft area or membrane protein cluster at the PSD [130–132]. Weakening or strengthening of this association via interface-targeting mutations, other than the R485L/F484A mutation, could affect the SynGAP docking onto the membrane and, thus, indirectly contribute to the disease state. The double mutation affects also the Rap1 activity [2], which is supported by the prior mutagenesis results showing that the SynGAP arginine finger is needed for Rap1-GTPase activity [26]. Despite a higher catalytic rate for Rap1, SynGAP exhibits higher affinity for Ras, which could be due to its RasGAP-family membership [8, 133].

Regarding the severity, one could argue that mutations disrupting the structure (Fig. 1B) and interactions at multiple levels, and/or showing destabilizing effects, are likely to be severely pathogenic (e.g. I494R). Still, the modelling approach could prove most dependable when annotating variants as benign (N = 53; Table S1); i.e. if the residue swap is not causing notable change (e.g. V343I, I529T, and S535T), the mutation could be well-tolerated. Nonetheless, caution should be exercised even with benign variants because the mutated residues could have further intra-protein contacts with parts missing from the model. Moreover, functional SynGAP is presumed to be a homotrimer, and the arrangement outside the coiled-coil domain is unknown [27].

In conclusion, this research provides a structural annotation of missense variants, supports the notion that extensive unfolding is not a prerequisite for SynGAP-associated NSID [4] and, importantly, reveals potential interactions between SynGAP, the intracellular membrane, and RasGTPase. These structural insights pave the way for improved clinical diagnosis and structure-based drug discovery for SynGAP.

**Key Points**

- The structural effects of missense mutations on SynGAP1 were explored using in silico mutagenesis and molecular modelling;
- The effects of missense mutations were probed using extensive atomistic molecular dynamics (MD) simulations (N = 211; 3 x 150 ns; totaling ~10 μs);
- The effects of missense mutations on the folding stability energy were probed using FoldX-MD and Rosetta calculations;
- Provides MD-based structural annotation for the SynGAP1 missense variants to assist clinical diagnosis;
- The first study to model SynGAP1 in complex with RasGTPase at the inner leaflet of post-synaptic membrane

## Supplementary Material

Supplementary_Information_bbae458

Table_S1_bbae458

supplementary_files

## Data Availability

The data used in these analyses is available upon request and, upon publication, will be provided free-of-charge via a dedicated online server.
