# Supplementary material for: Atomistic simulations reveal impacts of missense mutations on the structure and function of SynGAP1
Source: Brief Bioinform. 2024 Sep 23;25(6):bbae458. doi: 10.1093/bib/bbae458 (PMC11418247; doi:10.1093/bib/bbae458)
Supplement: Supplementary_Information_bbae458 [file supplementary_information_bbae458.pdf]

Supplementary Information

for

Atomistic Simulations Reveal Impacts of Missense Mutations on the Structure and Function of SynGAP1

Aliaa E. Ali[1,2], Li-Li Li[3], Michael J. Courtney[3], Olli T. Pentikäinen[1,2], Pekka A. Postila[1,2,*]

[1] MedChem.fi, Institute of Biomedicine, Integrative Physiology and Pharmacology, University of Turku, FI-20014 Turku, Finland

[2] InFLAMES Research Flagship, University of Turku, 20014 Turku, Finland

[3] Neuronal Signalling Laboratory and Turku Screening Unit, Turku Bioscience Centre, University of Turku and Åbo Akademi University, Turku, Finland

* Correspondence: pekka.postila@utu.fi

Methods

Protein Structure Preparation and In Silico Mutagenesis

The SynGAP model (AF-Q96PV0-F1-model_v3.pdb) was downloaded from the AlphaFold Protein Structure Database (https://alphafold.ebi.ac.uk/; accessed 22/01/2022) [1]. Based on the pLDDT, the ≥50 reliable parts (res. 198-364, 396-730) and less reliable Gly-rich Ω-loop (res. 365-395), were retained (Figure 1B-C). 211 missense variants, 204 from ClinVar (https://www.ncbi.nlm.nih.gov/clinvar/; accessed 21/04/2023) [2] and additional six from MASTERMIND (https://mastermind.genomenon.com/; accessed 21/04/2023) [2], were generated using MODELLER10.3 [3] (Table S1).

Modelling SynGAP in Complex with RasGTPase and Membrane

The SynGAP-membrane complex (Figure S2A) was modelled to facilitate WT system simulations at the inner post-synaptic membrane. The C2 domain of the model was aligned via the backbone C^{α} atoms against known Type I (RIM2; PDB: 2BWQ [4]) and Type II (PTEN; PDB: 1D5R [5]) C2 domain-containing structures acquired from the Protein Data Bank (PDB; https://www.rcsb.org/; accessed 11/10/2022) [6] using VERTAA in the BODIL [7] (Figure S2B). The upright C2 domain-membrane positioning (Figure 2A) was acquired by superposing the C2 domain against a protein (Figure S2C) with a settled membrane positioning at the OPM (Orientations of Proteins in Membranes; https://opm.phar.umich.edu/; accessed 11/10/2022; PDB: 4RJ9) [8,9]. The SynGAP-RasGTPase complex (Figure 3A), where the catalytic arginine finger binds to the Ras-bound substrate GTP (Figure 3B), was modelled by superposing the SynGAP-GAP domain against human GAP-RasGTPase complex (Figure 3C; PDB: 1WQ1) [10] and the C-terminal tail of Ras (res. 168-189) was taken from the AlphaFold model (AF-P01112-F1-model_v4.pdb; accessed 02/03/2023) [1]. The GDP-AlF_3 complex at the active site was modified to GTP in MAESTRO (Schrödinger Release 2022-3: Maestro, Schrödinger, LLC, New York, NY, 2022).

SynGAP-Solvent Molecular Dynamics Simulations

SynGAP-solvent molecular dynamics (MD) simulations were performed using AMBER22 [11]. The simulations were done for the WT protein (Figure 1A) and 211 variants (Table S1), each running for 3 x 150 ns. The system was prepared using TLEAP in AMBERTOOLS22 [12], the protein model was immersed in explicit water molecules (~32,500) within a 15 Å box radius. Na^+ counter ions were added for neutralization when necessary. The total system, containing ~138,400 atoms, utilized AMBERff19SB and OPC force fields for the protein and solvent, respectively [13,14].

Before the production MD simulations, a two-phase 2,000-step minimization, a 250-ps heating stage and a three-stage 250-ps equilibration were performed. The protein side chains and solvent were minimized before performing the unrestrained minimization on the entire system using the steepest descent method for four cycles followed by the full conjugate gradient minimization. The heating stage was performed in the canonical ensemble (NVT) where the system was heated to 310 K, whereas the equilibration stages were done using the isothermal-isobaric ensemble (NPT). During heating and equilibration, the restraint weight for the protein was lowered successively from 4 kcal/mol-$Å^2$ to

1. A time step of 2 fs and non-bonding cut-off of 10 Å were applied. The temperature and pressure were raised and maintained at 310 K and 1 bar using Langevin dynamics and isotropic Berendsen barostat [15,16], respectively. Electrostatic interactions were calculated with the Particle Mesh Ewald (PME) method [17], and hydrogen bond (or H-bond) lengths were constrained with the SHAKE algorithm [18]. The WT system was also simulated at 400 K to assess the model's thermal stability. The AMBER input files are given in the Supplementary Information (SI).

Before ensuing with the in silico mutagenesis, the WT system (Figure 1B) was pre-processed using the above-described multi-step MD protocol. The lowest total energy frame of three 100-ns WT replica production MD simulations was selected as the 3D template for the mutagenesis. This pre-processing was done to ensure that the WT system was well-equilibrated before proceeding with the mutagenesis, final system preparation, and the SynGAP-solvent simulation steps, including the three replicate 150-ns production simulations (Figure S4). Note that the WT system was re-processed similarly to the variant systems with multistep scheme but without the in silico mutagenesis step.

SynGAP-Membrane and SynGAP-Ras-Membrane Complex Molecular Dynamics Simulations

The WT SynGAP protein was also modelled in complex with the RasGTPase and membrane (Figure 3A) or only with the membrane (Figure S2A) using CHARMM-GUI (https://www.charmm-gui.org/; accessed 03/08/2023) and CHARMM36 ff [19]. A three-component membrane model, composed of dilinoleicphosphatidylcholine (N = 352), dilinoleicphosphatidylethanolamine (N = 480) and dilinoleicphosphatidylserine [20,21] (N = 192; Figure S3), was selected to represent the inner post-synaptic cell membrane leaflet. Ras was Cys-palmitoylated at residues 181 and 184 (Figures 3A and S3). The SynGAP-membrane complex (683,370 atoms; Figure S2A) was solvated in a rectangular box with water thickness of 60 Å using the TIP3P water molecules and a total of 193 Na^+ counter ions were added to neutralize the system charge. The SynGAP-RasGTPase-membrane complex (560,059 atoms; Figure 3A) was prepared with water thickness of 40 Å and neutralized with 201 Na^+ counter ions. For comparison, the membrane-only system (467,656 atoms, 192 Na^+) was also prepared.

These membrane-related systems (Figures S2A and 3A) were MD simulated using GROMACS2021 [22]. Firstly, the system was minimized with no constraints using a 5,000-step steepest-descent approach. Secondly, the system was equilibrated in two phases for 250 ps at 310 K with a 1 fs time step using NVT simulations. Thirdly, 2,000 ps and 2 fs time step NPT simulations were performed in four additional equilibration phases using semi-isotropic Berendsen thermostat and barostat. Finally, fourthly, a constraint-free production MD simulation with 2 fs time step was performed. The pressure was set to 1 bar and the temperature to 310 K utilizing the Parrinello-Rahman barostat [23] and Nosé-Hoover thermostat [24], respectively. The electrostatic interactions were calculated with the Particle Mesh Ewald (PME) method [17] using a 10 Å cut-off. The H-bond distances were constrained using the LINCS algorithm [25].

The WT SynGAP-Ras-membrane system preparation (Figure 3A) required extra steps due to inclusion of the Cys-palmitoylated lipids. A 50-ns production MD simulation was done after performing the multi-step equilibration

described above. Then, the covalently attached lipids of Ras were pulled into the membrane using a steered MD simulation. The lipids (C16 atoms) were pulled along the Z-direction with a 500-ps umbrella pulling using a distance increase of 0.01 nm ps^{-1} and a force constant of 2000 KJ mol^{-1} nm^{-2}. Lastly, an 80-ns production simulation was performed with the final system where the lipids anchored RasGTPase to the membrane; thus, adding up the simulation time to 130 ns. In contrast, the SynGAP-membrane (Figure S2A) and membrane only systems were simulated after the equilibration steps for 300 ns and 150 ns, respectively. The MD simulation input files are given in the SI.

Assessing Protein Stability Changes Induced by Missense Mutations

The relative free energy of folding (ΔΔG) for WT and missense variants of SynGAP was estimated using the empirical force field algorithm FoldX5.0 [26]. Firstly, water and ions were stripped away, and a representative snapshot frame was extracted as a PDB file for every nanosecond in the 150-ns WT SynGAP-solvent simulations using CPPTRAJ in AMBERTOOLS22 [12]. Secondly, the RepairPDB option in FoldX was used to optimize the 150 extracted structures via six energy minimization cycles using to ensure potential energy convergence. Thirdly, the BuildModel option in FoldX was used to generate 211 missense variants using each of the optimized WT snapshot structure as a 3D template and, finally, the energy difference between the variant and WT systems was calculated. The modified MD protocol for FoldX has been introduced in prior studies [27–32]. The Gibbs free energy difference or ΔΔG between WT and variant protein is calculated with the following formula (Eq. 1):

Eq. 1

$$\Delta\Delta G = \Delta G_{WT} - \Delta G_{variant} \ [kcal^{-1}]$$

where ΔG_{WT} stands for the free energy of the WT system and $\Delta G_{variant}$ for the free energy of the missense variant system. The exact energy terms are detailed in the study introducing the force field [33,34]. Notably, the individual ΔΔG values are only comparable between the WT and variant systems that have the same structural context used in the calculation. The estimate of ΔΔG for each variant was calculated by averaging across all 150 frames and, finally, the averages for the three replica trajectories were also averaged (Table S1).

As mutations close to zero free energy difference (ΔG ±2 kcal/mol) cannot be classified with confidence [30], the cut-off values were chosen to be as inclusive as possible while maximizing the accuracy of the predictions. The energy cut-offs that determine if the variant could impact folding were set to ≥2 kcal and ≤-2 kcal for the destabilizing and stabilizing mutations, respectively, adapted based on suggestions from prior studies [30,31,35]. Variants falling within the energy barrier range from -0.5 to 0.5 were considered potentially benign based on the calculated standard deviation of 0.46 kcal/mol [26] when comparing the results against the clinical data. Whereas variants falling in-between the energy-based categories were designated ambiguous.

Additionally, the ΔΔG between the WT-SynGAP and the missense variants were calculated using the Rosetta Cartesian ΔΔG protocol devised by Frenz et al. [36–40]. Rosetta protocols allow backbone flexibility and can generate and explore different conformational ensembles from a single starting structure, unlike FoldX that suffers from backbone rigidity during sampling [31,41]. The 0 ns WT-SynGAP snapshot structure from each replica simulation was first relaxed using the full-atom FastRelax protocol [42]. 50 repeats were applied and the lowest energy relaxed

structure was chosen for the further ΔΔG calculations with the missense variants [31,43]. ΔΔG values were reported as the lowest energy difference between the relaxed WT and the Rosetta-generated variant ΔG values calculated by ref2015 scoring function in the cartesian space from five prediction iterations for each individual run [44,45]. The energy values were converted from Rosetta Energy Units (REUS) to Kcal/mol using the scaling factor 2.94 [41,46]. The energy calculations were run using the caretsian2020_ref2015.yaml and converted using aggregate.yaml provided by the customizable PYTHON wrapper RosettaDDGPrediction available under GNU General Public License v3.0, (https://github.com/ELELAB/RosettaDDGPrediction) [41]. The same cut-offs were applied for the pathogenicity prediction as in the FoldX-MD protocol.

Figures

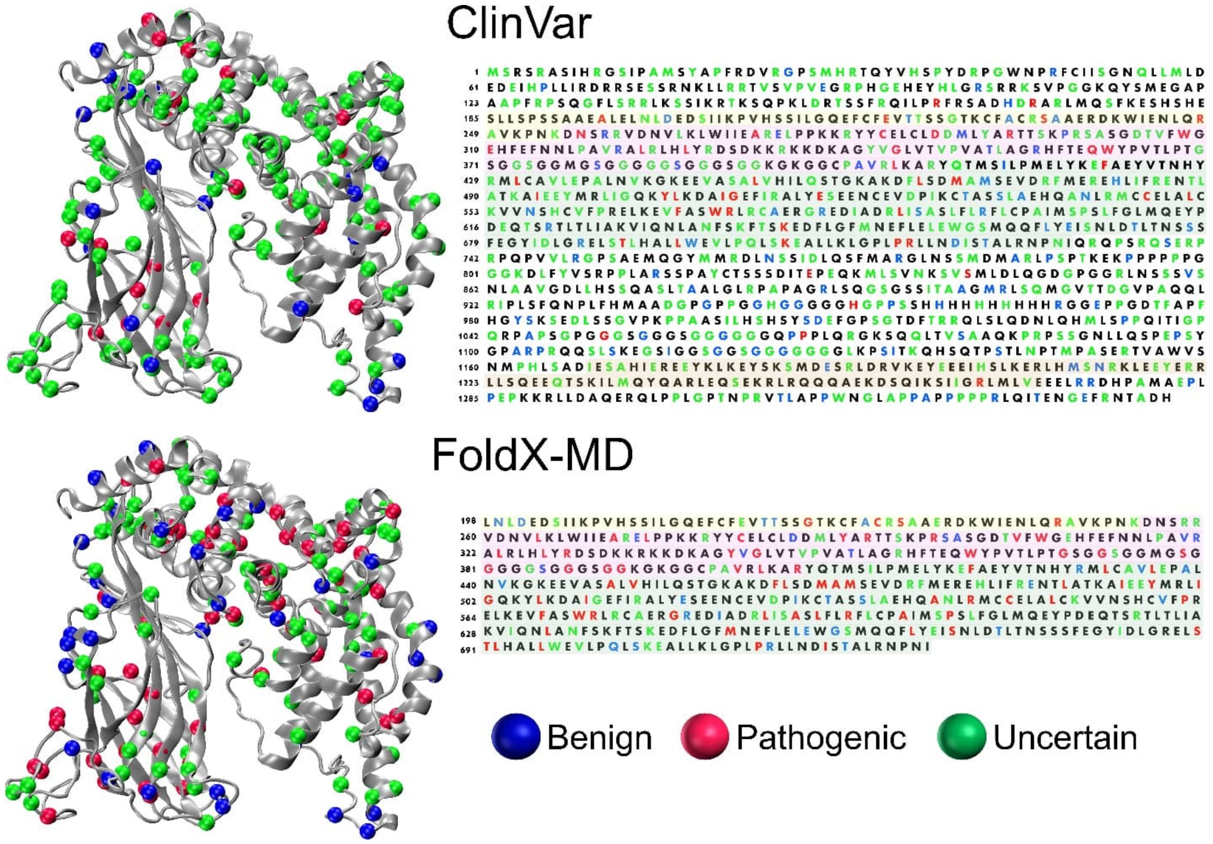

Figure S1. Missense mutation distribution with the SynGAP amino acid sequence and N-terminal model structure. On the top, the ClinVar assigned missense mutations are shown as spheres with the N-terminal protein 3D structure (res. 198-730; grey cartoon) and with the entire SynGAP α2 isoform 1 amino acid sequence (UniProt: Q96PV0-01). On the bottom, the missense mutation predictions are visualized similarly for the FoldX-MD against the N-terminal model and the matching amino acid sequence. The benign or likely benign (neutral; blue), pathogenic or likely pathogenic (destabilizing; red) and uncertain (VUS and conflicting; ambiguous; green) variants are assigned different colours, respectively. Note, that several of the residues have been reported to have multiple alternative mutations of differing status; however, the priority in the colouring was given to the pathogenic ones and then to the benign and uncertain ones. The PH (res. 198-255; yellow), C2 (res. 256-405; pink), GAP (res. 406-730; green) and CC (res. 1168-1270; brown) domains are highlighted with light colours in the sequences for reference.

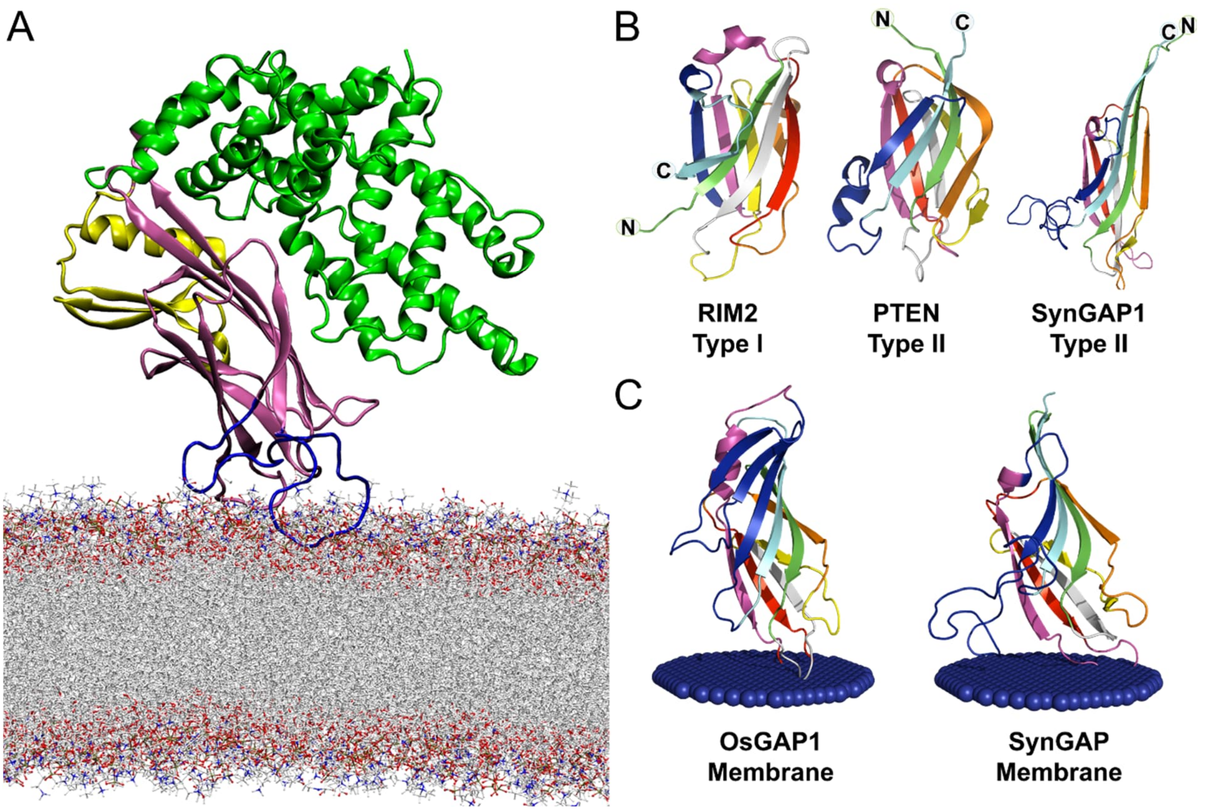

Figure S2. SynGAP-membrane complex. **(A)** SynGAP model (cartoon model) interacting via its C2 domain (magenta) with the post-synaptic inner leaflet membrane model (stick models) in the molecular dynamics (MD) simulation at 300 ns. The highly flexible Gly-rich **Ω** loop (blue) finds conformation that stabilizes the C2 domain-membrane association. **(B)** The C2 domains are arranged from two β sheets that are composed of eight β antiparallel strands, forming the β sandwich. This arrangement differs between Type I (e.g., RIM2 in PDB: 2BWQ; [4]) and Type II (e.g., PTEN in PDB: 1D5R [5]) C2 domains. The SynGAP C2 domain belongs to the Type II category. Matching β-strands between the C2 domains are painted with the same colours. **(C)** An established membrane orientation of the C2 domain from a plant GTPase activating protein 1 (OsGAP1; PDB: 4RJ9 [8,9]) was used as a basis for the positioning of the C2 domain of SynGAP (blue disc).

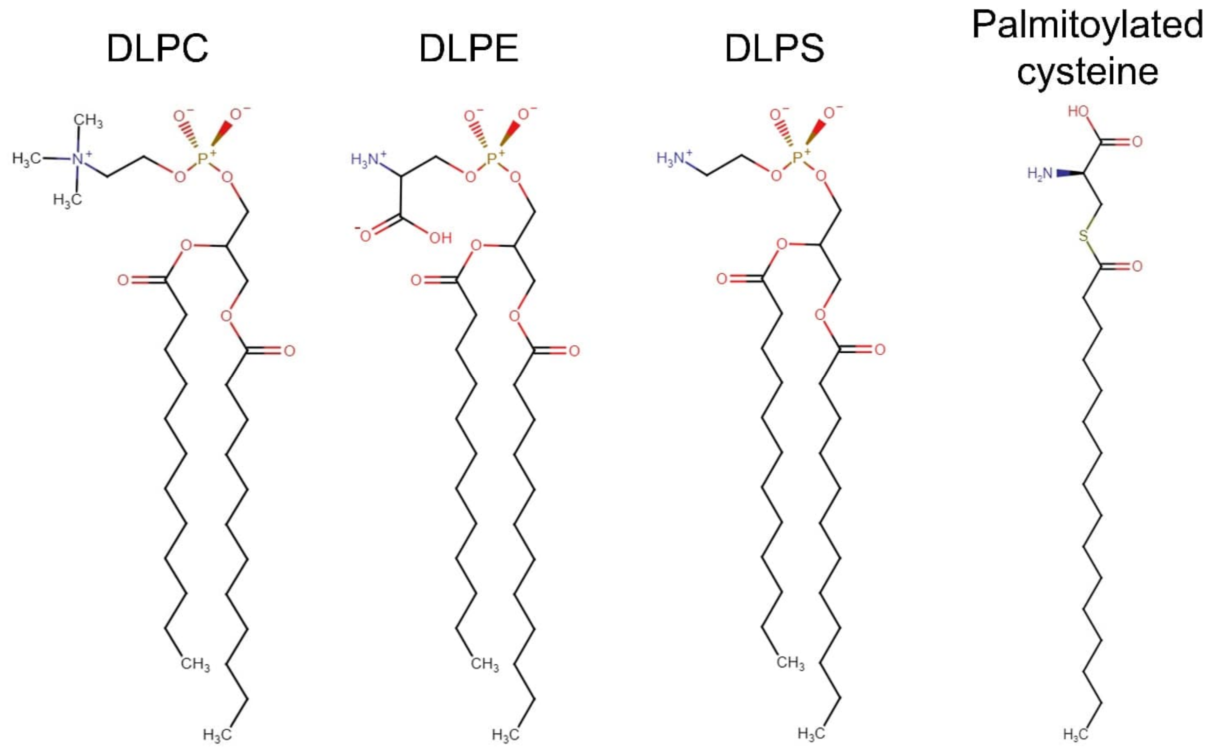

Figure S3. 2D structures of phospholipids and the covalently linked Cys-palmitoylated lipid groups used in the simulations. The phospholipids include dilinoleicphosphatidylcholine (DLPC), dilinoleicphosphatidylethanolamine (DLPE) and dilinoleicphosphatidylserine (DLPS). The 2D images, which were drawn online at the RCSB PDB (https://www.rcsb.org/chemical-sketch; accessed 05/10/2024) using Chemical Sketch Tool (ChemAxon20.20.0; http://www.chemaxon.com), were based on information from the CHARMM-GUI (https://www.charmm-gui.org/; accessed 03/08/2023) and CHARMM36 ff [19].

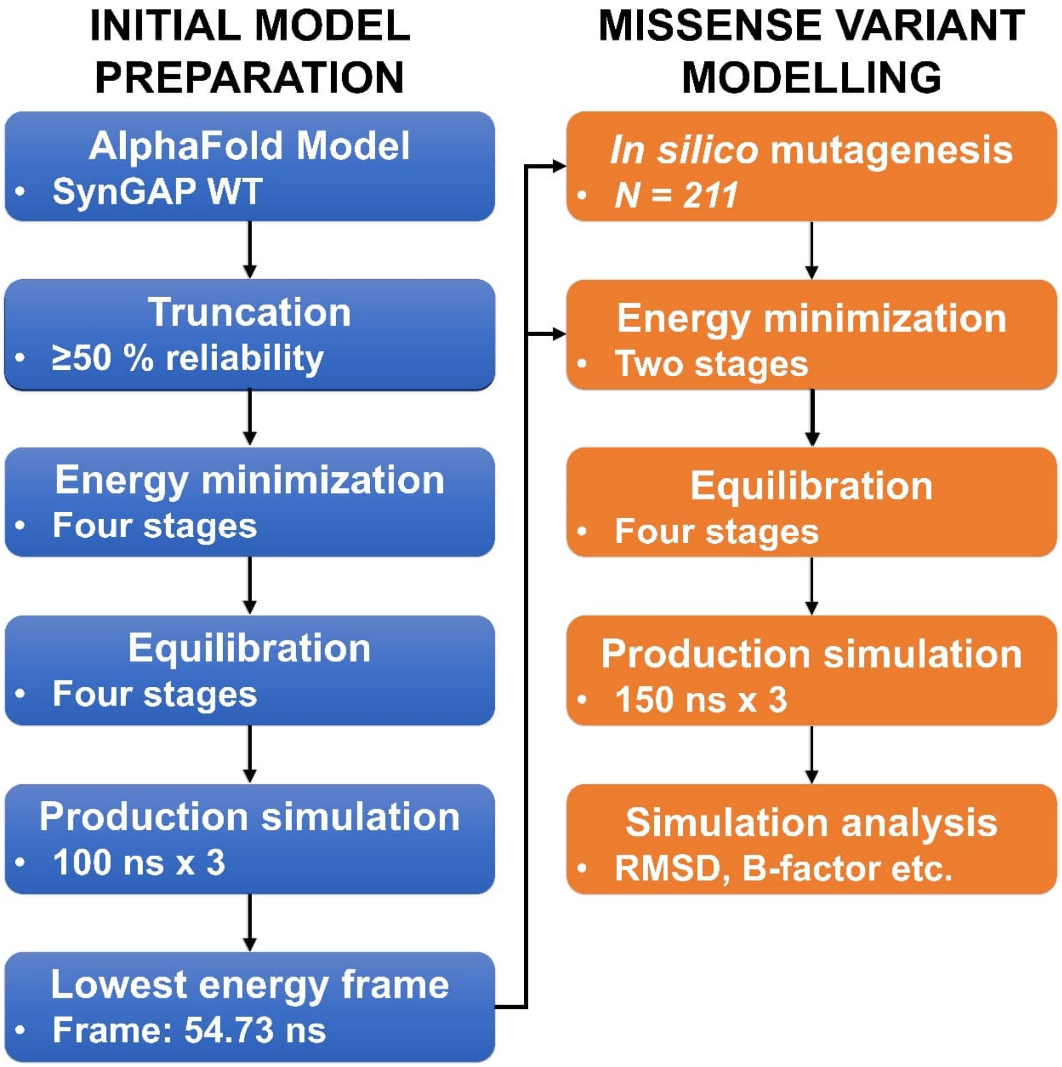

Figure S4. In silico mutagenesis and molecular dynamics simulation workflow for SynGAP missense variants in the solvent. Firstly, the solvated AlphaFold model, truncated to contain only the ≥50% reliable parts (res. 198-730), was energy minimised, heated and equilibrated under constraint simulations before performing the free 100-ns production MD simulations. Secondly, the lowest energy simulation frame was selected for performing the in silico mutagenesis and then repeating all of the simulation steps of the initial model preparation. Note that the WT system was prepared otherwise similarly as the missense variants but only the mutagenesis step was omitted.

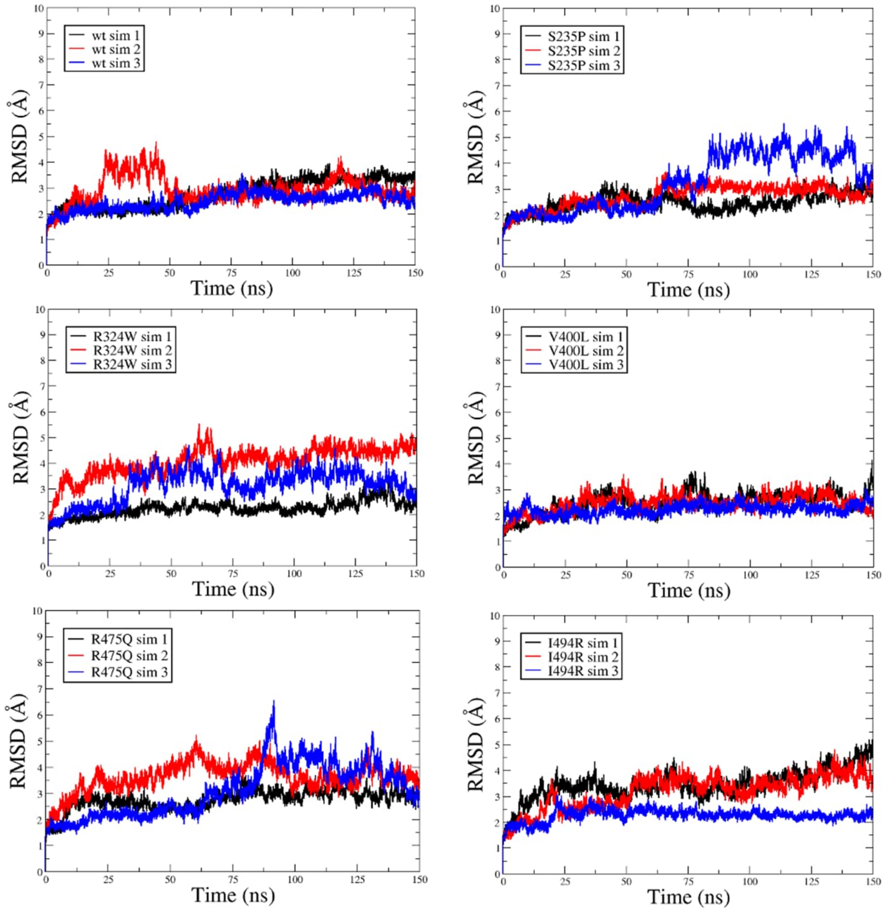

Figure S5. Root mean square deviation of protein backbone with a selected assortment of variants. The RMSD (Root Mean Square Deviation) was calculated for the entire 150 ns MD simulations using the first frame as a reference using CPPTRAJ in in AMBERTOOLS22 [12]. The RMSD values are plotted for the WT system and the missense variants, S235P (Likely Pathogenic), R324W (VUS), V400L (Benign), R475Q (Not in ClinVar) and I494R (Likely Pathogenic). The Ω Gly-rich loop was excluded from the calculations due to its excessive movement. Note that the replica simulations 1, 2 and 3 (black, red, and blue lines) may converge or end at slightly different RMSD levels, but based on systematic analysis this is not more frequent with either benign or pathogenic variants.

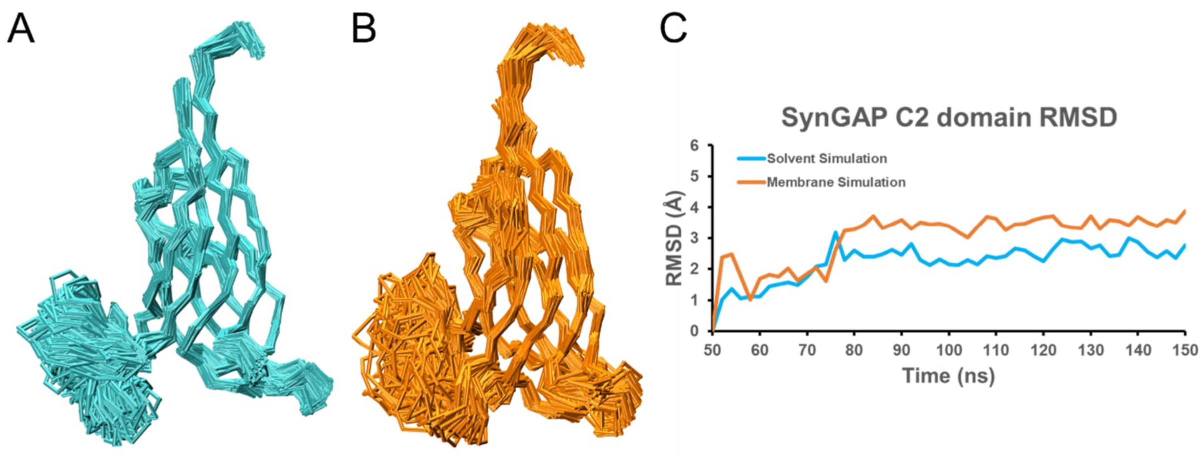

Figure S6. The effect of membrane on the SynGAP C2 domain stability and top loop movement. Two overlays of 100 MD simulation frames of the C2 domain structure are shown from **(A)** SynGAP solvent only simulation (cyan trace model) and **(B)** SynGAP-membrane complex (orange trace model) simulation. The frames, that were extracted between 50 to 150 ns (frame/ns), were aligned against the 50 ns frame using the protein backbone atoms in VMD 1.9.4a12 [47]. The Gly-rich Ω loop (residues 365-395) was excluded from the alignment due to its extreme volatility in both cases. **(C)** The RMSD calculations, which were done using CPPTRAJ in AMBERTOOLS22 [12] for the entire C2 domain, including also the Gly-rich Ω loop, indicate that the membrane association increases the C2 domain movement (orange line) and makes the membrane facing loop (Figure S2A) movement more dynamic in comparison to the solvent only simulation (blue line). In the solvent, the C2 domain loops can interact (e.g., H-bond, sterically stack) with each other stably, but with the membrane the loops are in constant interaction with the surface phospholipid head groups.

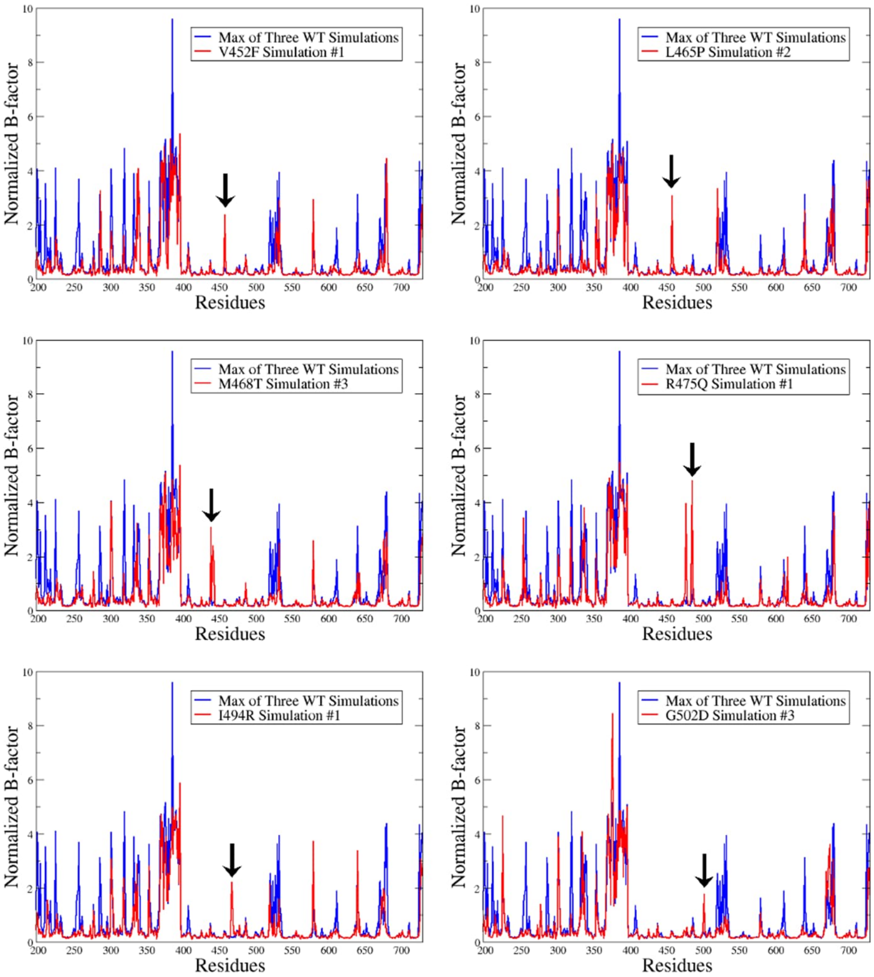

Figure S7. Normalized B-factors of protein backbone for a selected assortment of systems. The missense mutations prompt local changes in the SynGAP structure during the MD simulations, that are also reflected as increased fluctuation of the protein backbone. The normalized B-factors [48] were calculated for all frames included in the 150-ns production simulations. The normalized B-factors are shown with red lines for the following missense variants: V452F (VUS), L465P (Likely Pathogenic), M468T (VUS), R475Q (Not in ClinVar), I494R (Likely Pathogenic), and G502D (VUS). For comparison, the maximum normalized B-factor values of the three WT replica simulations were also plotted with blue lines. The extra peaks, caused by the mutations and not found in any of the replica WT simulations, are highlighted with black arrows focusing the attention on the local missense mutation-induced changes. For brevity and clarity, the results are only shown for one replica simulation where the effect of the mutation was apparent in the B-factors.

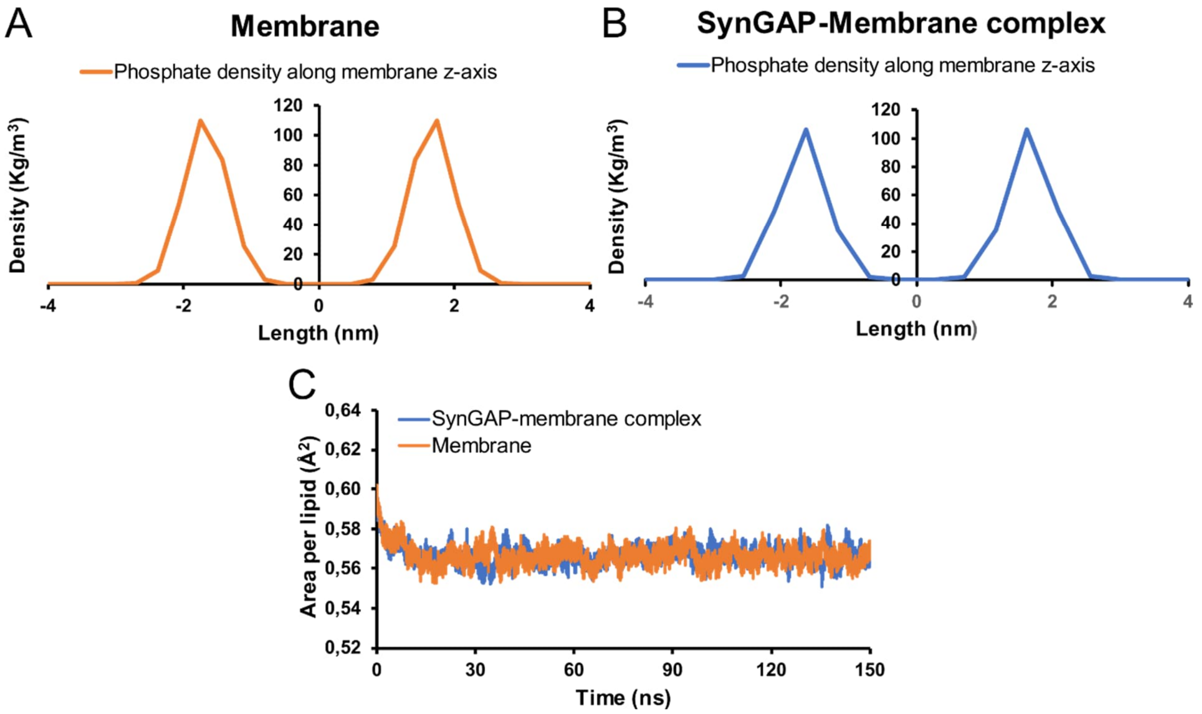

Figure S8. Lipid bilayer metrics for the membrane only and SynGAP-membrane systems in simulations. (A) The density curves for the phosphate atoms (orange line) indicate that the lipid bilayer thickness for the membrane only system was ~3.3 nm. **(B)** When the SynGAP protein was included to the membrane system, the bilayer thickness increased to ~3.5 nm based on the density curves (orange line). The thickness was calculated as the distance between the phosphate groups of the opposite leaflets using GMX DENSITY in GROMACS 2021 for the entire 150-ns and 300-ns simulation trajectory for A and B, respectively. **(C)** The membrane only (orange line) and SynGAP-membrane (blue line) systems generated similar area per lipid (APL) curves during the 150 ns simulations. The APL average value for the last 100 ns (50-150 ns) of the simulation trajectories was 0.57 for both the membrane only and SynGAP-membrane systems. The APL values were calculated based on the periodic boundary conditions (PBC) box X and Y coordinate changes using GMX ENERGY in GROMACS 2021.

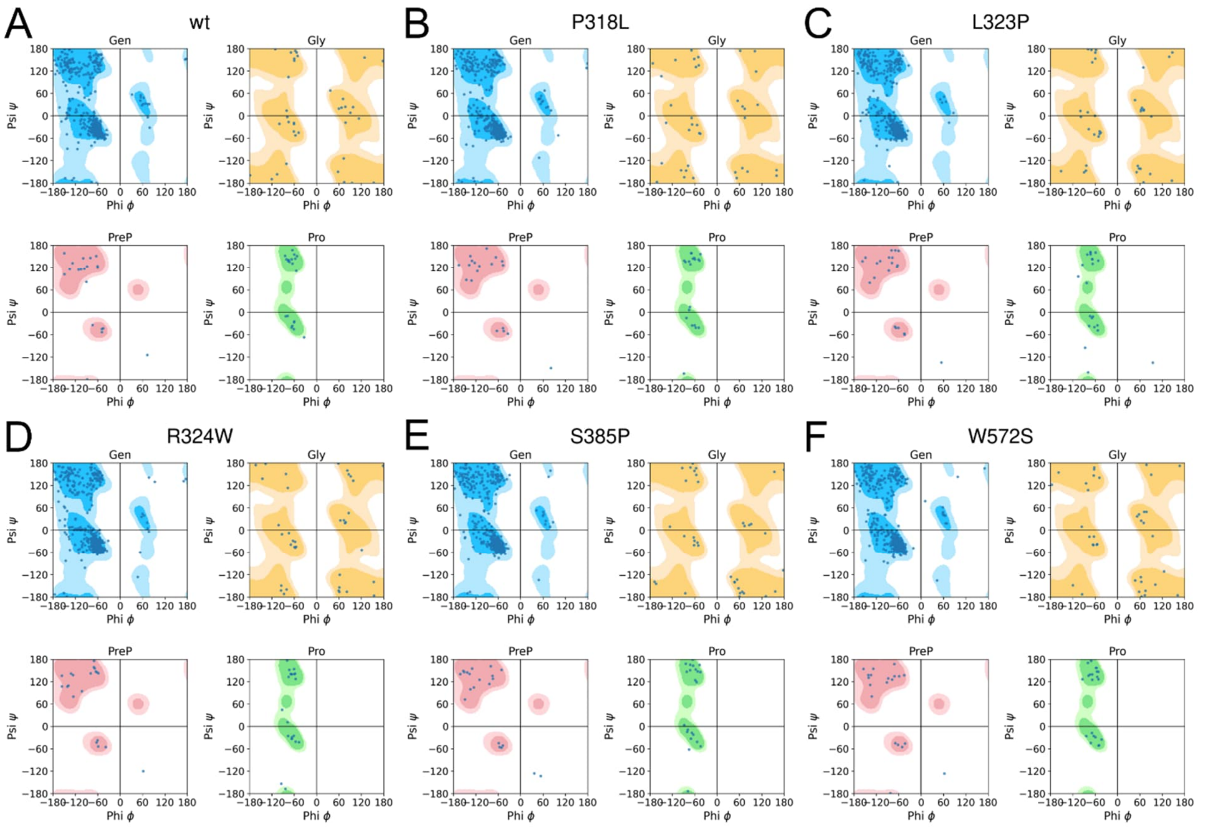

Figure S9. Selected assortment of Ramachandran plots at 150 ns from the SynGAP solvent only simulations. The Ramachandran plots generated using RAMAplot.py (GitHub: https://github.com/schlessinger-lab/RAMAplot; GNU Affero General Public License v3.0; accessed 01/11/2024) and CPPTRAJ in AMBERTOOLS22 [12], present a typical distribution of amino acid dihedral angles regardless of the severity or assigned pathogenicity status of the missense variant in question. The plotted systems include: **(A)** WT, **(B)** P318L (VUS), **(C)** L323P (not in ClinVar), **(D)** R324W (VUS), **(E)** S385P (VUS), and **(F)** W572S (Pathogenic). The plots are grouped into "Proline", "Pre-Proline" (one residue before Proline), "Glycine", and all other "General" amino acids to address different angle distributions of the amino acids. Note that the analysis was performed to all simulated SynGAP missense variants for 0 ns, 50 ns, 100 ns and 150 ns time steps, but the plots are shown here only for these six systems for brevity.

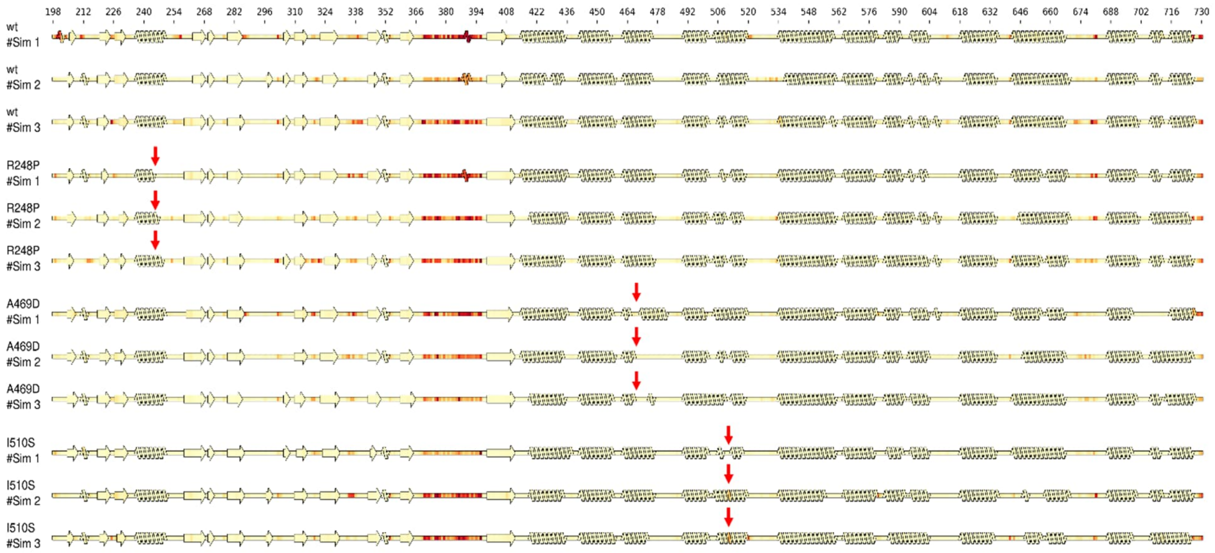

Figure S10. 2D protein secondary structure topology diagrams with B-factor colouring. The topologies, showing α helices (helical spring), β strands (arrows) and coils/loops/turns (lines), are shown for the last MD simulation frame or 150 ns snapshot using the 2D secondary structure diagrams. The colouring is done by normalized B-factor calculated over 150-ns MD simulation per residue [48]. The topologies were generated using SSDraw (GitHub: https://github.com/ethanchen1301/SSDraw) [49]. The diagrams are coloured with YlOrRd colormap in MATPLOTLIB using the side chain movement normalized B-factors calculated over the entire 150-ns simulations. In general, the substantial movement in the loops is indicated by the red colouring in all systems. The missense mutations did not generate consistent changes in the plotted secondary structure elements nor in the B-factor colouring over the WT system. However, the local changes induced by the mutations are indicated by the downward red arrows for the following systems: R248P (Likely Pathogenic), A469D (VUS), and I510S (Likely Pathogenic). Note that the analysis was performed to all 211 simulated SynGAP missense variants for 0 ns, 50 ns, 100 ns and 150 ns time steps, but the plots are shown only for these four systems for brevity.

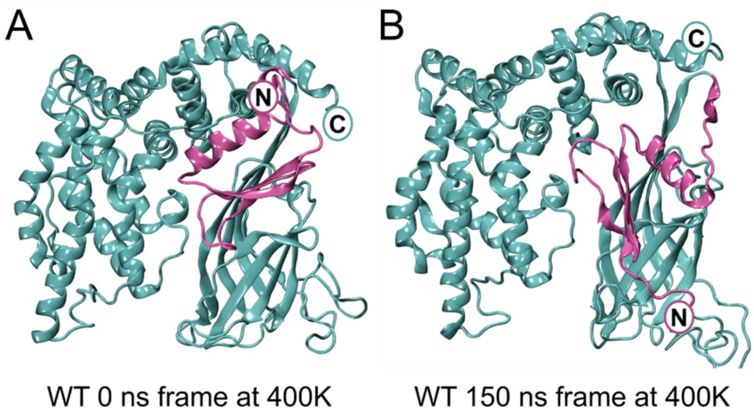

Figure S11. The N-terminal PH domain section rotates in 400 K molecular dynamics simulations. (A) The PH domain rotated from the initial WT position **(B)** to an alternative position in the 150-ns MD simulation performed at temperature of 400 K. The PH domain and the rest of the SynGAP model are shown as pink and cyan cartoons, respectively. The C- and N-termini of the model are highlighted with white spheres.

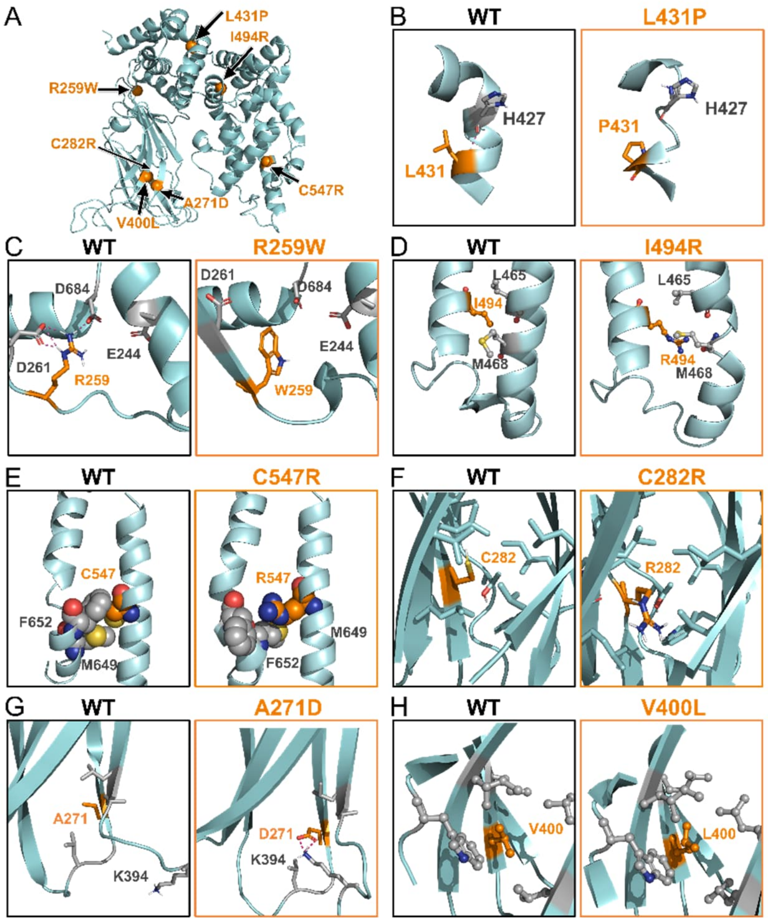

Figure S12. **Selected examples of SynGAP missense mutations**. **(A)** The location of the missense variants L431P, R259W, I494R, C547R, C282R, A271D, and V400L is shown (orange spheres) on the SynGAP model structure (cyan cartoon). **(B)** In the L431P variant, Pro431 cannot retain the same backbone H-bond with His431 (grey) on the α helix (res.427-438) as the leucine of the WT protein. **(C)** In the R259W variant, Trp259 cannot form the salt bridges with Asp261 of the C2 domain, or Asp684 of the GAP domain similarly as WT Arg259 is able to. **(D)** In the WT protein, Ile494 on the α helix (res. 489-519) packs hydrophobically with Met468 and Leu465 on the opposing α helix (res.460-477), while the large positive Arg494 side chain in the I494R variant disrupts the packing and weakens the helix integrity. **(E)** In the WT protein, Cys547 on the α helix (res.533-560) hydrophobically packs with Phe652 and Met649 on the opposing α helix (res. 641-666), while the large positive Arg547 disrupts this packing at the inter-helix space. **(F)** In the C282R variant, a large positively charged Arg282 is introduced into the hydrophobic core of the C2 domains, despite the implausibility of this scenario, only intra-protein distortion without large-scale unfolding or expulsion of the arginine from the core follows. **(G)** In the A271D variant, the carboxylate group of Asp271 on the β strand (res.259-272), unlike the methyl side chain of Ala271 in the WT protein, H-bonds with the nearby loop residues, thereby, interfering with the loop dynamics. **H)** Val400 and Leu400 on the β strand (res. 398-411) both in the WT and in the V400L variant systems, respectively, favourably pack inside a hydrophobic niche. 50 ns snapshots from MD simulations were used for the zoom-in views. The mutated residues are shown with orange backbone.

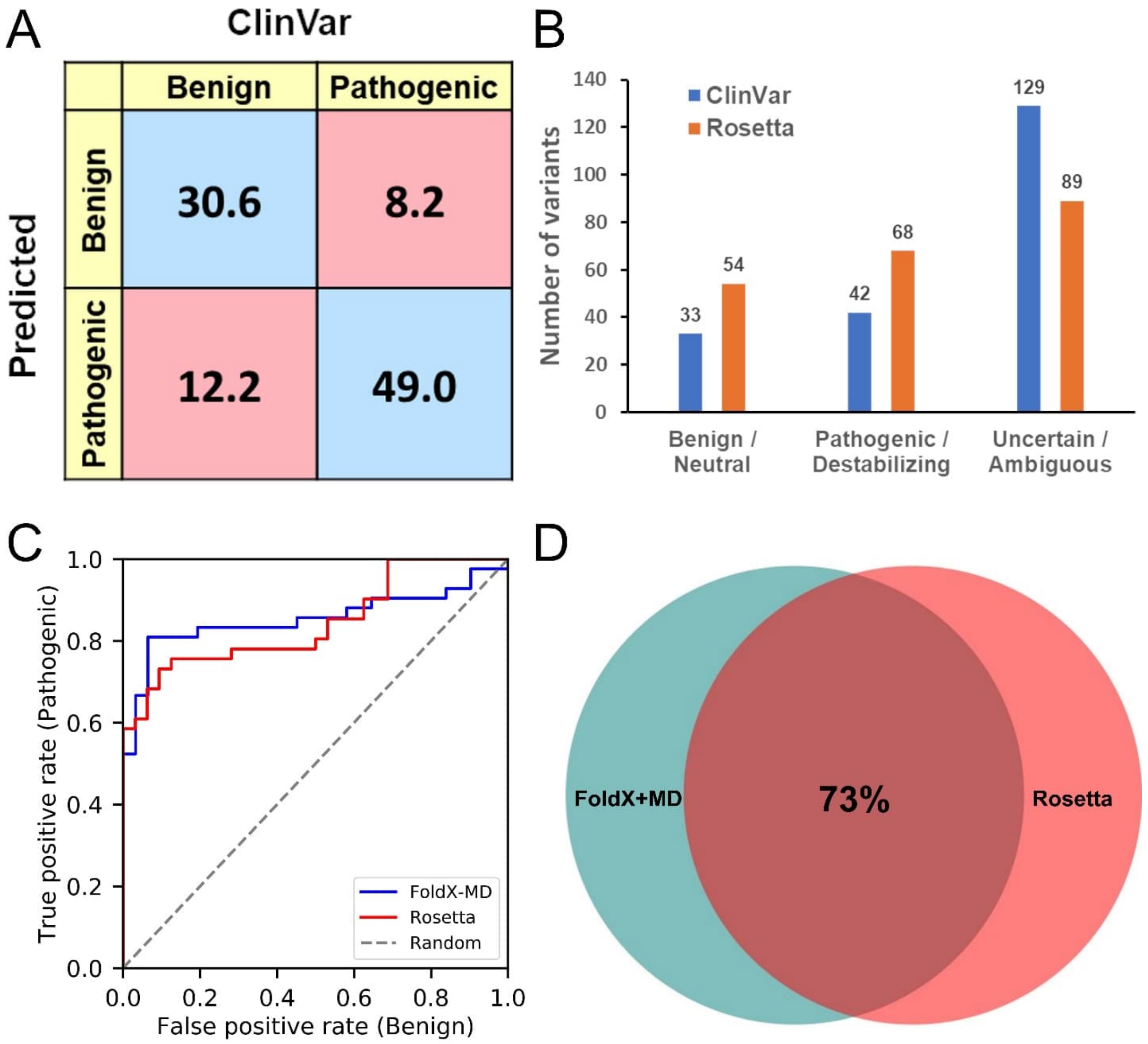

Figure S13. **Rosetta binding stability calculations of SynGAP missense mutations**. **(A)** The confusion matrix compares the neutral and destabilizing predictions of the Rosetta against the benign (or likely benign) and pathogenic (or likely pathogenic) variants from ClinVar. True positives and negatives are shown in blue, while false positives and negatives are in red. (**B**) The number of neutral predictions exceeds the known benign variants, yet more variants are classified as destabilizing than those identified as pathogenic. (**C**) ROC (Receiver Operating Characteristic) curves and AUC (Area Under Curve) values, that were calculated using ROCKER0.1.4 [50], show that Rosetta and FoldX-MD have comparable accuracy, albeit without statistically significant difference (Table S2). (**D**) The Venn diagram, that was drawn using DeepVenn (https://www.deepvenn.com/; accessed 10/07/2024) [51], indicates that predictions from FoldX-MD matched with ~73% of the Rosetta ones; excluding the ambiguous submissions. See Figure 5 for further details.

Table S2. The key hydrogen bonding residues of SynGAP in complex formation

SynGAP-membrane H-bonding			
**Residue (1)**	**H-bond % (3)**	**Mutations (4)**	**Nearby mutations (5)**
Lys336	20.6		D338G, R335H
Arg335	20.4	R335H	D338G
Arg329	19.8	R329H	L327P
Lys394	9.3		
Gly395	7.6		P398L
Lys278	7.3		R279W
Lys333	6.3		D338G
Ser371	6.0		S374Y
Lys277	5.8		G378D,R279W
Lys334	5.3		D338G
Asp332	4.7		
Lys392	3.1		G391V, G390E
**SynGAP-RasGTPase H-bonding**			
**Residue (2)**	**H-bond %**	**Mutations**	**Nearby mutations**
Arg485	75.8	R485C	E486K, L489F, L489P
Asp527	41.1	D527Y	I529T
Lys642	39.8	K642T	
Ile483	29.4		H481Y
Asn635	24.9	N635S	L633P
Arg485	22.2	R485C	E486K, L489F, L489P
Lys628	20.8		I630V
Lys442	11.3	K642T	
Lys492	10.5		I494R
Ser641	9.9		K642T
Leu595	7.8		R596C, L593H, R596H, R596L
His481	7.7	H481Y	
Glu486	6.2	E486K	R485C, L489F, L489P
Tyr614	5.3		
Ser302	4.8		S300F, A301T
Asn487	4.2		R485C, E486K, L489F, L489P
Arg499	3.9		Y497C, M498R, I501T, G502D
Glu490	3.9		L489F, L489P
Ser604	3.1	S604L	V400E, V400L, L402R

The H-bonding was analysed with CPPTRAJ in AMBERTOOLS22 [12]

(1) Residues H-bonding with membrane lipids throughout 300 ns SynGAP-membrane complex MD simulation.

(2) Residues H-bonding with RasGTPase throughout 130 ns SynGAP-RasGTPase-membrane complex MD simulation.

(3) The fraction of the production MD simulation the H-bond forms is given as percentage. Only the most prevalent H-bond per residue is considered (e.g., HE atom of Arg485), although the same residue could form multiple H-bonds involving different atoms (e.g., HH12 atom of Arg485) simultaneously or separately. Only those residues forming H-bonds for at least three percent of the simulation time are included.

(4) SynGAP missense mutations for the reported H-bonding residue from the 211 mutations probed in the study.

(5) SynGAP missense mutations close to the reported H-bonding residue from the 211 mutations probed in the study.

Table S3. The accuracy of pathogenicity prediction methods with SynGAP missense ClinVar data

Prediction method [1]	Precision [2]	Recall [3]	Accuracy [4]	AUC [5]
**Pathogenic as positive**				
FoldX-MD	0.94	0.89	0.87	0.85±0.04
Rosetta	0.80	0.86	0.80	0.84±0.05
**Benign as positive**				
**Prediction method [1]**	**Precision [2]**	**Recall [3]**	**Accuracy [4]**	**AUC [5]**
FoldX-MD	0.71	0.83	0.87	0.85±0.05
Rosetta	0.79	0.71	0.80	0.84±0.05

The predictions are given either regarding benign or pathogenic variants from ClinVar [52], respectively, as positives. Note that benign and likely benign variants are combined similarly as pathogenic and likely pathogenic ones.

(1) The prediction methods include: Rosetta and FoldX-MD [26,37–39].

(2) Precision is a metric that tells how often the positive predictions are correct.

$$Precision = \frac{\Sigma\, TP}{\Sigma\, TP + FP}$$

(3) Recall is a metric that tells how often the method identifies positive instances correctly (TP) from all the actual positive cases.

$$Recall = \frac{\Sigma\, TP}{\Sigma\, TP + FN}$$

(4) Accuracy is a metric that tells how often correct predictions are made.

$$Accuracy = \frac{\Sigma\, TP + TN}{\Sigma\, TP + FP + FN + TN}$$

References

1. Jumper J, Evans R, Pritzel A, et al. Highly accurate protein structure prediction with AlphaFold. Nature 2021; 596:583–589

2. Chunn LM, Nefcy DC, Scouten RW, et al. Mastermind: A Comprehensive Genomic Association Search Engine for Empirical Evidence Curation and Genetic Variant Interpretation. Front Genet 2020; 11:

3. Šali A, Blundell TL. Comparative Protein Modelling by Satisfaction of Spatial Restraints. J Mol Biol 1993; 234:779–815

4. Dai H, Tomchick DR, García J, et al. Crystal Structure of the RIM2 C2A-Domain at 1.4 Å Resolution,. Biochemistry 2005; 44:13533–13542

5. Lee J-O, Yang H, Georgescu M-M, et al. Crystal Structure of the PTEN Tumor Suppressor: Implications for Its Phosphoinositide Phosphatase Activity and Membrane Association. Cell 1999; 99:323–334

6. Berman HM, Westbrook J, Feng Z, et al. The Protein Data Bank. Nucleic Acids Res 2000; 28:

7. Lehtonen J V, Still D-J, Rantanen V-V, et al. BODIL: a molecular modeling environment for structure-function analysis and drug design.

8. Yung YL, Cheung MY, Miao R, et al. Site-directed mutagenesis shows the significance of interactions with phospholipids and the G-protein OsYchF1 for the physiological functions of the rice GTPase-activating protein 1 (OsGAP1). Journal of Biological Chemistry 2015; 290:23984–23996

9. Lomize MA, Lomize AL, Pogozheva ID, et al. OPM: orientations of proteins in membranes database. Bioinformatics 2006; 22:623–625

10. Scheffzek K, Ahmadian MR, Kabsch W, et al. The Ras-RasGAP Complex: Structural Basis for GTPase Activation and Its Loss in Oncogenic Ras Mutants. Science (1979) 1997; 277:333–339

11. Case DA, Aktulga HM, Belfon K, et al. Amber 2023, University of California, San Francisco.

12. Case DA, Aktulga HM, Belfon K, et al. AmberTools. J Chem Inf Model 2023; 63:6183–6191

13. Tian C, Kasavajhala K, Belfon KAA, et al. ff19SB: Amino-Acid-Specific Protein Backbone Parameters Trained against Quantum Mechanics Energy Surfaces in Solution. J Chem Theory Comput 2020; 16:528–552

14. Izadi S, Anandakrishnan R, Onufriev A V. Building water models: A different approach. Journal of Physical Chemistry Letters 2014; 5:3863–3871

15. Loncharich RJ, Brooks BR, Pastor RW. Langevin dynamics of peptides: The frictional dependence of isomerization rates of N-acetylalanyl-N′-methylamide. Biopolymers 1992; 32:523–535

16. Berendsen HJC, Postma JPM, van Gunsteren WF, et al. Molecular dynamics with coupling to an external bath. J Chem Phys 1984; 81:3684–3690

17. Darden T, York D, Pedersen L. Particle mesh Ewald: An N·log(N) method for Ewald sums in large systems. J Chem Phys 1993; 98:10089–10092

18. Miyamoto S, Kollman PA. Settle: An analytical version of the SHAKE and RATTLE algorithm for rigid water models. J Comput Chem 1992; 13:952–962

19. Brooks BR, Brooks CL, Mackerell AD, et al. CHARMM: The biomolecular simulation program. J Comput Chem 2009; 30:1545–1614

20. Van Meer G, Voelker DR, Feigenson GW. Membrane lipids: Where they are and how they behave. Nat Rev Mol Cell Biol 2008; 9:112–124

21. Postila PA, Vattulainen I, Róg T. Selective effect of cell membrane on synaptic neurotransmission. Sci Rep 2016; 6:

22. Lindahl, Abraham, Hess, et al. GROMACS 2021 Source code. 2021;

23. Parrinello M, Rahman A. Crystal Structure and Pair Potentials: A Molecular-Dynamics Study. Phys Rev Lett 1980; 45:1196–1199

24. Hoover WG, Holian BL. Kinetic moments method for the canonical ensemble distribution. Phys Lett A 1996; 211:253–257

25. Hess B, Bekker H, Berendsen HJC, et al. LINCS: A Linear Constraint Solver for molecular simulations. J Comput Chem 1997; 18:1463–1472

26. Schymkowitz J, Borg J, Stricher F, et al. The FoldX web server: An online force field. Nucleic Acids Res 2005; 33:

27. Yu H, Wang M jun, Xuan N xia, et al. Molecular dynamics simulation of the interactions between EHD1 EH domain and multiple peptides. J Zhejiang Univ Sci B 2015; 16:883–896

28. Miller CR, Johnson EL, Burke AZ, et al. Initiating a watch list for Ebola virus antibody escape mutations. PeerJ 2016; 2016:

29. Gonzalez TR, Martin KP, Barnes JE, et al. Assessment of Software Methods for Estimating Protein-Protein 2 Relative Binding Affinities.

30. Sapozhnikov Y, Patel JS, Ytreberg FM, et al. Statistical modeling to quantify the uncertainty of FoldX-predicted protein folding and binding stability. BMC Bioinformatics 2023; 24:

31. Barnes JE, Lund-Andersen PK, Patel JS, et al. The effect of mutations on binding interactions between the SARS-CoV-2 receptor binding domain and neutralizing antibodies B38 and CB6. Sci Rep 2022; 12:

32. Chitongo R, Obasa AE, Mikasi SG, et al. Molecular dynamic simulations to investigate the structural impact of known drug resistance mutations on HIV-1C Integrase-Dolutegravir binding. PLoS One 2020; 15:

33. Schymkowitz JWH, Rousseau F, Martins IC, et al. Prediction of water and metal binding sites and their affinities by using the Fold-X force field. PNAS 2005; 102:

34. van Durme J, Delgado J, Stricher F, et al. A graphical interface for the FoldX forcefield. Bioinformatics 2011; 27:1711–1712

35. Potapov V, Cohen M, Schreiber G. Assessing computational methods for predicting protein stability upon mutation: Good on average but not in the details. Protein Engineering, Design and Selection 2009; 22:553–560

36. Frenz B, Lewis SM, King I, et al. Prediction of Protein Mutational Free Energy: Benchmark and Sampling Improvements Increase Classification Accuracy. Front Bioeng Biotechnol 2020; 8:

37. Leaver-Fay A, Tyka M, Lewis SM, et al. Rosetta3: An object-oriented software suite for the simulation and design of macromolecules. Methods Enzymol 2011; 487:545–574

38. Fleishman SJ, Leaver-Fay A, Corn JE, et al. Rosettascripts: A scripting language interface to the Rosetta Macromolecular modeling suite. PLoS One 2011; 6:

39. Chaudhury S, Lyskov S, Gray JJ. PyRosetta: A script-based interface for implementing molecular modeling algorithms using Rosetta. Bioinformatics 2010; 26:689–691

40. Leman JK, Weitzner BD, Lewis SM, et al. Macromolecular modeling and design in Rosetta: recent methods and frameworks. Nat Methods 2020; 17:665–680

41. Sora V, Laspiur AO, Degn K, et al. RosettaDDGPrediction for high-throughput mutational scans: From stability to binding. Protein Science 2023; 32:

42. Tyka MD, Keedy DA, André I, et al. Alternate states of proteins revealed by detailed energy landscape mapping. J Mol Biol 2011; 405:607–618

43. Valanciute A, Nygaard L, Zschach H, et al. Accurate protein stability predictions from homology models. Comput Struct Biotechnol J 2023; 21:66–73

44. Alford RF, Leaver-Fay A, Jeliazkov JR, et al. The Rosetta All-Atom Energy Function for Macromolecular Modeling and Design. J Chem Theory Comput 2017; 13:3031–3048

45. Shapovalov M V., Dunbrack RL. A smoothed backbone-dependent rotamer library for proteins derived from adaptive kernel density estimates and regressions. Structure 2011; 19:844–858

46. Park H, Bradley P, Greisen P, et al. Simultaneous Optimization of Biomolecular Energy Functions on Features from Small Molecules and Macromolecules. J Chem Theory Comput 2016; 12:6201–6212

47. Humphrey W, Dalke A, Schulten K. VMD: Visual molecular dynamics. J Mol Graph 1996; 14:33–38

48. Fuchs JE, Waldner BJ, Huber RG, et al. Independent metrics for protein backbone and side-chain flexibility: Time scales and effects of ligand binding. J Chem Theory Comput 2015; 11:851–860

49. Chen EA, Porter LL. SSDraw: software for generating comparative protein secondary structure diagrams.

50. Lätti S, Niinivehmas S, Pentikäinen OT. Rocker: Open source, easy-to-use tool for AUC and enrichment calculations and ROC visualization. J Cheminform 2016; 8:

51. Hulsen T. DeepVenn-a web application for the creation of area-proportional Venn diagrams using the deep learning framework Tensorflow.js.

52. Landrum MJ, Lee JM, Benson M, et al. ClinVar: improving access to variant interpretations and supporting evidence. Nucleic Acids Res 2018; 46:D1062–D1067
